# Population genetic characteristics of *Aedes aegypti* in 2019 and 2020 under the distinct circumstances of dengue outbreak and the COVID-19 pandemic in Yunnan Province, China

**DOI:** 10.3389/fgene.2023.1107893

**Published:** 2023-03-09

**Authors:** Ge Wang, Jian Gao, Zu Ma, Yuan Liu, Ming Wang, Dan Xing, Chunxiao Li, Xiaoxia Guo, Teng Zhao, Yuting Jiang, Yande Dong, Hengduan Zhang, Tongyan Zhao

**Affiliations:** State Key Laboratory of Pathogen and Biosecurity, Beijing Institute of Microbiology and Epidemiology, Beijing, China

**Keywords:** *Aedes aegypti*, genetic diversity, haplotype, molecular marker, China

## Abstract

**Introduction:** Since *Aedes aegypti* invaded Yunnan Province in 2002, its total population has continued to expand. Shi et al. used microsatellite and mitochondrial molecular markers to study the *Ae. aegypti* populations in Yunnan Province in 2015 and 2016, found that it showed high genetic diversity and genetic structure. However, there are few studies on the population genetic characteristics of *Ae. aegypti* in Yunnan Province under different levels of human intervention. This study mainly used two common types of molecular markers to analyze the genetic characteristics of *Ae. aegypti*, revealing the influence of different input, prevention and control pressures on the genetic diversity and structure of this species. Understanding the genetic characteristics of *Ae. aegypti* populations and clarifying the diversity, spread status, and source of invasion are essential for the prevention, control and elimination of this disease vector.

**Methods:** We analyzed the genetic diversity and genetic structure of 22 populations sampled in Yunnan Province in 2019 and 17 populations sampled in 2020 through nine microsatellite loci and COI and ND4 fragments of mitochondrial DNA. In 2019, a total of 22 natural populations were obtained, each containing 30 samples, a total of 660 samples. In 2020, a total of 17 natural populations were obtained. Similarly, each population had 30 samples, and a total of 510 samples were obtained.

**Results:** Analysis of *Ae. aegypti* populations in 2019 and 2020 based on microsatellite markers revealed 67 and 72 alleles, respectively. The average allelic richness of the populations in 2019 was 3.659, while that in 2020 was 3.965. The HWE analysis of the 22 populations sampled in 2019 revealed significant departure only in the QSH-2 population. The 17 populations sampled in 2020 were all in HWE. The average polymorphic information content (PIC) values were 0.546 and 0.545, respectively, showing high polymorphism. The average observed heterozygosity of the 2019 and 2020 populations was 0.538 and 0.514, respectively, and the expected average heterozygosity was 0.517 and 0.519, showing high genetic diversity in all mosquito populations. By analyzing the COI and ND4 fragments in the mitochondrial DNA of *Ae. aegypti*, the populations sampled in 2019 had a total of 10 COI haplotypes and 17 ND4 haplotypes. A total of 20 COI haplotypes were found in the populations sampled in 2020, and a total of 24 ND4 haplotypes were obtained. STRUCTURE, UPGMA and DAPC cluster analyses and a network diagram constructed based on COI and ND4 fragments showed that the populations of *Ae. aegypti* in Yunnan Province sampled in 2019 and 2020 could be divided into two clusters. At the beginning of 2020, due to the impact of COVID-19, the flow of goods between the port areas of Yunnan Province and neighboring countries was reduced, and the sterilization was more effective when goods enter the customs, leading to different immigration pressures on *Ae. aegypti* population in Yunnan Province between 2019 and 2020, the source populations of the 2019 and 2020 populations changed. Mantel test is generally used to detect the correlation between genetic distance and geographical distance, the analysis indicated that population geographic distance and genetic distance had a moderately significant correlation in 2019 and 2020 (2019: *p* < 0.05 R2 = 0.4807, 2020: *p* < 0.05 R2 = 0.4233).

**Conclusion:** *Ae. aegypti* in Yunnan Province maintains a high degree of genetic diversity. Human interference is one reason for the changes in the genetic characteristics of this disease vector.

## Introduction

*Aedes aegypti* originated in Africa (Brown et al., 2014) and spread to other continents with the slave trade ships between the 15th and 17th centuries, after which it invaded Asia in the late 19th century (Smith, 1956).The main distribution areas of *Ae. aegypti* include tropical and subtropical regions (Tabachnick, 1991). It is the main vector of dengue virus (DENV) (Simmons et al., 2012), yellow fever virus (Jentes et al., 2011) and chikungunya virus (CHIKV) (Leparc-Goffart et al., 2014). In the past, it was believed that the distribution of *Ae. aegypti* in China included only in the areas south of 22° north latitude (Shi et al., 2017a). Since the discovery of *Ae. aegypti* at Jiegao Port, Yunnan Province, in 2002 (Xueshu et al., 2004), the population of *Ae. aegypti* has shown an expansion trend in Yunnan Province. In a 2015 survey, *Ae. aegypti* was found in seven counties/cities in Yunnan Province, namely, Jinghong, Mengla, Menghai, Yingjiang, Longchuan, Ruili, and Lushui (Yang et al., 2015).

Yunnan Province is in southwestern China, with subtropical and tropical monsoon climates, which provides favorable conditions for the breeding and transmission of mosquitoes. Therefore, there are many kinds of mosquitoes with wide distribution and high density, which is conducive to the maintenance and transmission of existing arboviruses and the emergence of new arboviruses (Zha et al., 2012; Hameed et al., 2020). The Southeast Asian countries (Myanmar, Laos, and Vietnam) bordering Yunnan Province are all dengue fever endemic areas (Zhang et al., 2013). Dengue fever is an acute infectious disease transmitted by *Ae. aegypti* and *Ae. albopictus* (Shi et al., 2017b)*.* It is one of the main public health problems in the world. Dengue fever is mainly prevalent in tropical and subtropical areas, and one-third of the world’s people are at risk of infection (Chen and Vasilakis, 2011). Before 2000, in Yunnan Province, dengue fever cases had been reported only in Hekou County in 1975 (Zhang et al., 1999). In 2008, local cases of dengue fever were reported for the first time in Mangshi, Dehong Prefecture, Zhenkang County, and Lincang City (Li et al., 2010). Since then, dengue fever cases have been reported every year. In 2013, a number of dengue fever outbreaks occurred in Xishuangbanna and Dehong (Li et al., 2016; Yuanyuan et al., 2016). According to a survey of dengue fever outbreaks in recent years, a high density of *Ae. aegypti* was monitored at the outbreak sites. Detection and survey results for *Ae. aegypti* populations also showed that the distribution areas were consistent with the local dengue fever epidemic area. This phenomenon means that in recent years, *Ae. aegypti* has become the main transmission vector of DENV in Yunnan Province (Shi and Zhao, 2016).

Biological invasion is closely related to human health and genetic factors of invasive species, such as genetic variation and diversity (Ivey et al., 2019). These factors are important indicators for revealing the status of invasive species (Rasheed et al., 2013; Chown et al., 2015). After an invasion, the invasive species may establish in the new habitat; eventually, the invasion of the species leads to ecological imbalance in the local area, which changes or destroys the local ecological environment and severely reduces biodiversity (Mollot et al., 2017). Identifying invasions and spread is critical for improving prevention and control measures.

With the emergence of molecular biology technology, abundant genetic markers have been identified for the study of population genetic characteristics. Microsatellite markers are widely used in population genetics research, especially in the exploration of invasive biological populations, taking advantage of their ability to analyze invasion routes, diffusion paths, evolutionary conditions and population genetic structure characteristics of invading populations (Cao et al., 2015; Maynard et al., 2017; Lv et al., 2020). Such analyses revealed that *Ae. aegypti* in Brazil reinvaded from northern South America and the Caribbean after being completely eliminated based on analysis of *Ae. aegypti* in Brazil using 12-site microsatellite markers (Monteiro et al., 2014). Pless et al. (2017) used microsatellite markers to trace the invasion of *Ae. aegypti* in California, United States, and clarified that *Ae. aegypti* in California may come from the central and southern regions of the United States . Microsatellite loci can also be used to evaluate the genetic variation and population structure of mosquitoes at a fine scale. Zhang et al. used nine pairs of microsatellite markers to study *Ae. albopictus* in Nanjing, China, and found that it can be divided into two clades (Zhang et al., 2022).

Mitochondrial DNA has a wealth of genetic information, mainly because of its maternal inheritance. Its advantages are a simple structure, fast evolution, and abundant molecular markers. Therefore, mitochondrial DNA has become one of the most widely used sources of molecular markers for determining species gene flow, and it is frequently used in population genetic studies, particularly in *Ae. aegypti* populations (Feng et al., 2016; Abuelmaali et al., 2022). Naim et al. analyzed the COI fragments of the mitochondrial DNA of *Ae. aegypti* in Penang, Malaysia, and found 39 haplotypes. The genetic variation within populations was low, but that between populations was high, and overall population differentiation wasn’t obvious (Naim et al., 2020). Gao et al. (2021) used COI gene to classify *Ae. albopictus* in different climatic regions of China and found that it was divided into two clades, with three dominant haplotypes.

In this study, microsatellite markers and mitochondrial DNA markers were used to analyze the genetic characteristics of natural *Ae. aegypti* populations in Yunnan Province sampled in 2019 and 2020. In 2019, a total of 5,371 local dengue cases were reported in Yunnan Province, compared with 237 cases in 2020. The main reason for this decrease was that beginning in 2020, due to the prevention and control measures related to COVID-19, people and goods entering through customs along the border between China and Laos and between China and Myanmar drastically declined (Wei et al., 2021). The introduction of *Ae. aegypti* thus underwent tremendous changes between 2019 and 2020. The degree of genetic structure and variability may depend on the success of national vector control campaigns, migration and international trade flows from each country (Escobar et al., 2022). This study aimed to compare the invasion and spread routes, population diversity and population structure characteristics of *Ae. aegypti* in different years to clarify the changes in its population genetic characteristics under different mosquito control and immigrations and provide suggestions for the local epidemic prevention department to formulate future prevention and control measures.

## Materials and method

### Mosquito sampling

From September to October of 2019 and 2020, we collected *Ae. aegypti* larvae in Xishuangbanna Prefecture (Mengla County, Jinghong City, Menghai County), Lincang City (Nansan Town, Mengding Town), and Dehong Prefecture (Ruili City). To avoid inbreeding effects, we selected at least six larvae from each breeding site in a designated area within 100 m and mixed the larval mosquitoes. All the collected mosquitoes were brought back to the laboratory and separated by sampling site for rearing until they grew into adults, and then they were identified through analysis of morphological characteristics under a microscope to confirm that they were all *Ae. aegypti* (LBL, 1999). All adult *Ae. aegypti* samples were preserved in 100% ethanol at 4°C for the isolation of genomic DNA.

### DNA isolation and PCR amplification

Genomic DNA was extracted individually from the samples using the Qiagen DNeasy Blood and Tissue Kit (no. 69504, Qiagen, Germany) following the standard DNA extraction protocol provided by the manufacturer. The eluted DNA was measured by a spectrophotometer and stored in a freezer at −40°C for later use.

In this study, nine microsatellite sites developed by Shi were used for analysis (Shi et al., 2017b). The detailed primer information is shown in the Supplementary Appendix SA1. All PCRs were performed on a T100 Thermal Cycler (Bio-Rad, United States) in a 50 μL reaction system containing 0.25 U PrimeSTAR HS DNA polymerase (10 pmol/μL, TaKaRa, Japan), 6 μM dNTPs (2.5 mM each, TaKaRa, Japan) and 5 μM each primer and 10 ng DNA. The PCR program was set as follows: 35 cycles of 95°C for 30 s, 57°C for 30 s and 72°C for 1 min followed by a final elongation at 72°C for 10 min. All products were then checked with 1.2% agarose gel electrophoresis under UV light. After confirming that they were the target band, they were run on a 3730XL DNA analyzer (Applied Biosystems, United States).

We used COI and ND4 gene markers of mitochondrial DNA to analyze the genetic structure of *Ae. aegypti* populations in Yunnan Province. With the primer set 5′-GGA​GGA​TTT​GGA​AAT​TGA​TTA​GTT​C-3′ (F-COI) and 5′- CCCGGTAAAATTAAAATATAAACTTC-3′(R-COI) plus 5′- ATT​GCC​TAA​GGC​TCA​TGT​AG -3′ (F-ND4) and 5′- TCG​GCT​TCC​TAG​TCG​TTC​AT-3′ (R-ND4), DNA amplification of 550 bp fragments of COI and 361 bp fragments of ND4 was performed on a T100 Thermal Cycler (Bio-Rad, United States). The reaction system was 25 μL, which included 5 µL PCR buffer (TaKaRa, Japan), 4 µL dNTPs (2.5 mM each, TaKaRa, Japan), 1 µL primers (10 pmol/μL, TaKaRa, Japan), 0.5 µL PrimeSTAR HS DNA polymerase (TaKaRa, Japan) and 15.75 µL ddH_2_0. The PCR amplification programs were set as follows: predenaturation at 94°C for 2 min, followed by 40 cycles of denaturation at 94°C for 60 s, annealing at 55°C for 30 s and elongation at 72°C for 1 min, with a final elongation at 72°C for 10 min (COI gene), and predenaturation at 94°C for 2 min, followed by 35 cycles of denaturation at 94°C for 60 s, annealing at 52°C for 30 s and elongation at 72°C for 1 min, with a final elongation at 72°C for 10 min (ND4 gene). All PCR products were detected by 1.2% agarose gel electrophoresis and sequenced on an ABI 3730XL automatic sequencer (Applied Biosystems, United States).

### Microsatellite analysis of population genetic characteristics

To study the population genetic characteristics of *Ae. aegypti*, some software programs were used to calculate the standard genetic parameters. All microsatellite markers were examined for polymorphism by determining their polymorphic information content (PIC) values *via* PIC-Calc version 0.6 (Shao et al., 2013). The number of alleles (NA) was calculated by Cervus version 3.0.7 (Zhang et al., 2016). In each population, the allelic richness (r) was assessed by FSTAT (version 2.9.3.2) (Cheng et al., 2006). The observed heterozygosity (Ho), expected heterozygosity (He), inbreeding coefficient (Fis) and fixation index (Fst) values for each population were evaluated with the analysis of molecular variance (AMOVA) test in Arlequin version (version 3.5.2.2) (Excoffier et al., 2005). Departures from Hardy–Weinberg equilibrium (HWE) was assessed *via* Arlequin (version 3.5.2.2) and the *p*-value was obtained through multiple comparison corrections. The null allele frequency of each microsatellite locus was calculated by Micro-Checker software (version 2.2.3) (Oosterhout et al., 2010). The stepwise mutation model (SMM) and two-phase model of mutation (TPM) were used to test for bottleneck effects *via* Bottleneck software (version 1.2.0.2) (Men et al., 2013). STRUCTURE software implementing Bayesian clustering analysis was used to assess the genetic structure differences among populations (Pritchard et al., 2000). The parameters were set as follows: 200,000 burn-in periodand 1,000,000 sampling period. The K value was set from 1 to 24 for the populations sampled during 2019 and 1 to 19 for the populations sampled in 2020. The optimal K value was assessed *via* STRUCTURE HARVESTER (Earl and Vonholdt, 2012). After that, the differences among the populations were displayed in graphs using DISTRUCT (version 1.1) (Rosenberg, 2004). Discriminant analysis of principal components (DAPC) for the populations was performed with the R package “Adegenet 2.1.3” (Jombart, 2008). Isolation-by-distance (IBD) analysis was performed between genetic distance [FST/(1-FST)] and geographical distance [ln(km)], a Mantel test was performed *via* NTSYS (version 2.2), and then graphs of the correlations between geographic distance and genetic distance were generated in Excel 2019 (Microsoft Corporation, WA, United States).

### Mitochondrial DNA analysis of the genetic characteristics of *Ae. aegypti*

DNASP 5.0 software was used to calculate the genetic diversity parameters of the haplotypes, including haplotype diversity (Hd), nucleotide diversity (Pi) and average number of nucleotide differences (π) (Rozas et al., 2003). The genetic relationships between all haplotypes were displayed through Network (version 5.0.1.1) (Ahlswede et al., 2000). Arlequin (version 3.5.2.2) software was used to perform neutrality tests based on Tajima’s D and Fu’s Fs.

## Results

A total of 22 locations were sampled from September to October 2019, including nine locations at Jinghong City, one location in Mengla County, one location at Daluo Port, one location at Mohan Port, three locations at Ruili City, one location at Nansan Town, Zhenkang County, three locations at Mengding Town, two locations at Qingshuihe Port and one location at Myanmar Muse.

**FIGURE 1:**
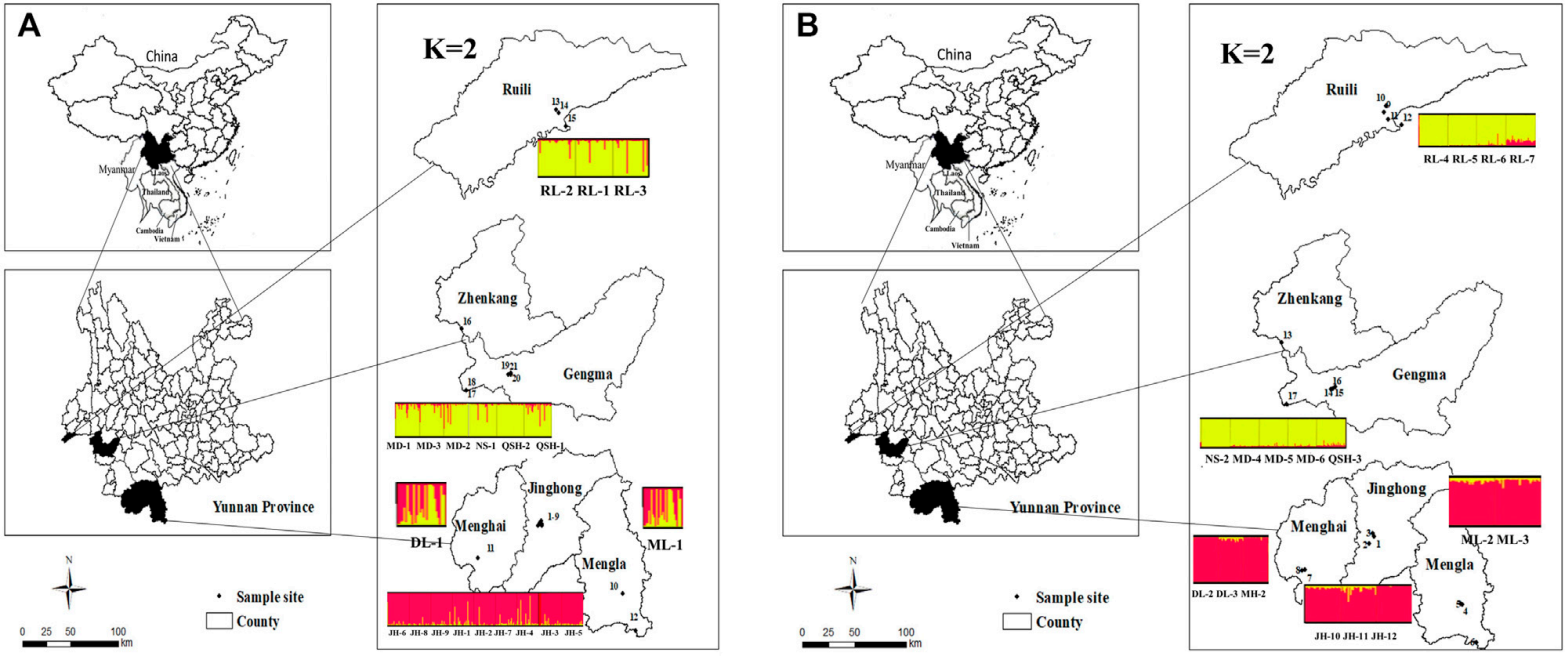
Distribution map of sampling locations and a plot of K = 2 cluster in Yunnan Province. Structure bar plot for all *Ae. aegypti* populations used in this study. The height of each color represents the probability of assignment to a specific cluster Figure 1 **(A)**: Distribution map of sampling locations and a plot of K = 2 cluster in Yunnan Province, 2019. Figure 1 **(B)**: Distribution map of sampling locations and a plot of K = 2 cluster in Yunnan Province, 2020.

In 2020, a total of 17 locations were sampled from September to October in Yunnan Province, including three locations at Jinghong City, two locations at Mengla County, two locations at Daluo Port, one location at Mohan Port, four locations in Ruili City, one location at Nansan Town, three locations at Mengding Town, and one location at Qingshuihe Port. Detailed acquisition information was listed in the Table 1 and Figure 1.

**TABLE 1:** Detailed information on the field sampling sites in Yunnan Province, 2019 and 2020.

Year	Collection region	No.	Location name	Code	No. Samples	Geographical coordinates	Collection
							Date
2019	Jinghong City	1	Suantang Restaurant	JH-1	30	N22°00′38.1″E100°48′13.5″	10/2019
		2	Brother Auto Market	JH-2	30	N21°59′54.3″E100°46′13.3″	10/2019
		3	Prefecture Committee Compound②	JH-3	30	N22°00′34.3″E100°47′54.1″	10/2019
		4	Prefecture Committee Compound①	JH-4	30	N22°00′31.4″E100°47′56.0″	10/2019
		5	Prefecture Transportation Company	JH-5	30	N22°01′16.6″E100°47′20.7″	10/2019
		6	Dalirenjia restaurant	JH-6	30	N21°59′51.2″E100°48′25.8″	10/2019
		7	Yipinchuan restaurant	JH-7	30	N21°59′44.7″E100°48′28.1″	10/2019
		8	Kunman Community	JH-8	30	N22°01′56.4″E100°47′51.8″	10/2019
		9	Manjinglan road	JH-9	30	N22°00′04.1″E100°48′29.5″	10/2019
	Mengla Town	10	Xinping Commissary	ML-1	30	N21°28′21.1″E101°34′02.9″	09/2019
	Daluo Port	11	Mengban Village	DL-1	30	N21°44′54.0″E100°12′19.1″	09/2019
	Mohan Port	12	Yunyu Hotel	MH-1	30	N21°11′19.2″E101°41′21.9″	09/2019
	Ruili City	13	91 Non-gken Road	RL-1	30	N24°00′56.0″E97°51′53.2″	09/2019
		14	Jinquan Road Nanmao Lake Park	RL-2	30	N24°00′32.8″E97°52′14.1″	09/2019
		15	Feibo Road	RL-3	30	N23°59′01.1″E97°53′15.2″	09/2019
	Nansan Town	16	Zhiyuan Auto Repair Factory	NS-1	30	N23°45′08.5″E98°48′59.9″	09/2019
	Qingshuihe Port	17	China-Myanmar Border Trade Market	QSH-1	30	N23°28′49.0″E98°50′26.7″	09/2019
		18	Yunxiangyuan restaurant	QSH-2	30	N23°28′51.6″E98°50′27.8″	09/2019
	Mengding Town	19	Hongwei express	MD-1	30	N23°33′27.1″E99°05′00.5″	09/2019
		20	Xiangyuexiaozhu Restaurant	MD-2	30	N23°33′09.0″E99°04′54.5″	09/2019
		21	Landfill	MD-3	30	N23°33′03.0″E99°04′05.9″	09/2019
	Myanmar	22	Myanmar Muse	MD1	30	N23° 59′50″E97° 56′21.3″	09/2019
2020	Jinghong City	1	Xiaojungan restaurant	JH-10	30	N22°00′34.4″E100°48′15.7″	09/2020
		2	Team 4 of Subtropical Crops Research Institute	JH-11	30	N22°01′57″E100°47′20″	09/2020
		3	Gasa town	JH-12	30	N21°57′21″E100°45′30″	09/2020
	Mengla Town	4	Supply and Marketing Cooperative	ML-2	30	N21°29′0″E101°33′52″	09/2020
		5	Hongfu Community	ML-3	30	N21°29′30″E101°33′22″	09/2020
	Daluo Port	6	Mengban Village	DL-2	30	N21°45′3″E100°12′14″	09/2020
		7	Manhong Village	DL-3	30	N21°44′46″E100°11′2″	09/2020
	Mohan Port	8	Western District	MH-2	30	N21°11′36″E101°41′19″	09/2020
	Ruili City	9	Huafeng Market	RL-4	30	N24°00′39″E97°51′48″	09/2020
		10	Munao Road	RL-5	30	N24°1′25.46″E97°52′2″	09/2020
		11	Non-gsha Road	RL-6	30	N23°59′51″E97°52′16″	09/2020
		12	China-Myanmar Friendship Road	RL-7	30	N23°59′10″E97°54′3.24″	09/2020
	Nansan Town	13	Xilin Village	NS-2	30	N23°45′22″E98°49′2″	09/2020
	Mengding Town	14	Hantai Tire Shop	MD-4	30	N23°33′6″E99°3′43″	09/2020
		15	Hongwei Express	MD-5	30	N23°33′37″E99°4′58″	09/2020
		16	Binhai Steel Wholesale Department	MD-6	30	N23°33′19″E99°4′14″	09/2020
	Qingshuihe Port	17	Hongteng restaurant 5	QSH-3	30	N23°29′3″E98°50′26″	09/2020

### Microsatellite analysis of population diversity

In this study, the above nine pairs of primers were used to amplify 660 samples collected in 2019 and 510 samples collected in 2020.

Through the analysis of microsatellite markers of *Ae. aegypti* samples collected in 2019 and 2020, a total of 67 and 72 alleles were obtained, respectively. On average, the 2019 populations obtained 7.44 alleles per locus, and the 2020 populations obtained eight alleles per locus. In 2019, SQM6 obtained 14 alleles, which was the highest number among all loci. The SQM5 and SQM9 loci had four alleles, which was the lowest number among all loci, while in 2020, the SQM2 and SQM6 loci had the most alleles (14), and SQM5 and SQM9 had only four alleles. The PIC values of the populations sampled in 2019 and 2020 were generally high, with average values of 0.546 and 0.545, respectively. Microchecker software revealed null alleles at nine loci in the 2 years, with frequencies ranging from 0.001 to 0.159, however, frequency *p* values <0.2 are considered to have no significant effect on the accuracy of data analysis by many studies (Dakin and Avise, 2004; Chapuis and Estoup, 2007) (Table 2).

**TABLE 2:** Polymorphic information at nine microsatellite loci in 2019 and 2020.

Year	Locus	No.	Number of alleles	PIC	Null allele frequency
2019	SQM1	660	6	0.407	0.112
	SQM2	660	13	0.763	0.047
	SQM3	660	8	0.489	0.151
	SQM4	660	6	0.18	0.057
	SQM5	660	4	0.486	0.004
	SQM6	660	14	0.765	0.006
	SQM7	660	5	0.647	0.032
	SQM8	660	7	0.646	0.068
	SQM9	660	4	0.535	0.007
	MEAN (2019)	660	7.44	0.546	0.054
2020	SQM1	510	6	0.445	0.032
	SQM2	510	14	0.783	0.047
	SQM3	510	9	0.567	0.159
	SQM4	510	6	0.165	0.075
	SQM5	510	4	0.427	0.001
	SQM6	510	14	0.831	0.049
	SQM7	510	7	0.626	0.024
	SQM8	510	8	0.562	0.036
	SQM9	510	4	0.501	0.007
	MEAN (2020)	510	8	0.545	0.048

We conducted the Hardy–Weinberg equilibrium test with significance *p* < 0.05 for the populations in 2019 and 2020. The HWE analysis of the 22 populations sampled in 2019 revealed significant departure only in the QSH-2 population. The 17 populations sampled in 2020 were all in HWE (Table 3).

**TABLE 3:** Locus information of each population in 2019 and 2020.

Year	Population	No.	Number of alleles	SI	Ho	He	HWE
							(*p*-value)
2019	JH-1	30	4.000	1.040	0.558	0.586	0.238
	JH-2	30	4.000	0.989	0.512	0.542	0.109
	JH-3	30	3.444	0.901	0.601	0.519	0.257
	JH-4	30	3.778	1.057	0.563	0.592	0.085
	JH-5	30	4.111	1.091	0.578	0.601	0.158
	JH-6	30	4.000	1.030	0.670	0.569	0.360
	JH-7	30	4.111	0.997	0.557	0.536	0.321
	JH-8	30	3.778	0.929	0.568	0.511	0.196
	JH-9	30	4.000	1.007	0.595	0.548	0.183
	ML-1	30	3.667	0.798	0.473	0.456	0.282
	DL-1	30	3.111	0.759	0.530	0.445	0.441
	MH-1	30	2.111	0.578	0.507	0.381	0.183
	RL-1	30	3.000	0.721	0.459	0.430	0.324
	RL-2	30	3.889	0.880	0.407	0.470	0.295
	RL-3	30	4.222	0.843	0.497	0.454	0.221
	NS-1	30	3.556	1.805	0.533	0.466	0.191
	MD-1	30	4.111	1.008	0.526	0.552	0.205
	MD-2	30	3.667	0.908	0.541	0.512	0.353
	MD-3	30	4.111	0.945	0.464	0.520	0.449
	QSH-1	30	4.778	1.074	0.586	0.575	0.384
	QSH-2	30	3.222	0.933	0.570	0.549	0.030 *
	MD1	30	4.889	1.072	0.548	0.561	0.295
	Mean	30	3.798	0.971	0.538	0.517	0.263
2020	JH-10	30	4.556	1.090	0.616	0.570	0.123
	JH-11	30	4.222	1.108	0.576	0.609	0.261
	JH-12	30	4.444	0.956	0.475	0.509	0.430
	ML-2	30	4.111	0.865	0.500	0.480	0.395
	ML-3	30	4.333	1.008	0.541	0.532	0.333
	DL-2	30	3.222	0.646	0.327	0.361	0.460
	DL-3	30	3.778	0.898	0.515	0.489	0.161
	MH-2	30	3.333	0.864	0.545	0.492	0.343
	RL-4	30	4.778	0.958	0.478	0.488	0.377
	RL-5	30	5.000	1.065	0.519	0.549	0.389
	RL-6	30	4.556	0.991	0.463	0.529	0.265
	RL-7	30	5.444	1.150	0.573	0.584	0.347
	NS-2	30	3.778	0.811	0.552	0.459	0.225
	MD-4	30	4.778	1.014	0.515	0.545	0.240
	MD-5	30	4.333	1.018	0.591	0.559	0.486
	MD-6	30	4.222	0.938	0.448	0.511	0.195
	QSH-3	30	5.111	1.093	0.504	0.555	0.282
	Mean	30	4.353	0.969	0.514	0.519	0.312

*** : *p* < 0.001; **: *p* < 0.01; *: *p* < 0.05.

In the 2019 populations, 157 of 792 pairwise tests for linkage disequilibrium remained significant after Bonferroni correction, while 166 of 612 pairwise tests for linkage disequilibrium remained significant in the 2020 populations. No consistency was found between any pair of loci in the 2 years. Details were listed in Supplementary Appendix SA2.

The genetic diversity of *Ae. aegypti* within the 22 populations sampled in 2019 showed that the observed heterozygosity was higher than the expected heterozygosity, indicating that the *Ae. aegypti* populations in Yunnan Province included some foreign individuals (Table 3).

The results of genetic diversity analysis of the 17 populations sampled in 2020 showed that the observed heterozygosity was lower than the expected heterozygosity, indicating inbreeding (Table 3).

In this study, Fstat software was used to analyze the allelic richness of 22 populations sampled in 2019 and 17 populations sampled in 2020 (Table 4). The 2019 populations were divided into four regions. Among them, the three populations collected in Xishuangbanna, except that in Jinghong City, showed the lowest allelic richness (3.11), and the Myanmar population had the highest allelic richness, which was 4.62. The statistical test (one-way ANOWA) of Myanmar population and populations in the border area of Yunnan Province showed that the *p* = 0.023, indicating that the allelic richness of Myanmar population was higher than that of Yunnan populations. The 2020 populations were also classified into four regions. The four populations in Ruili City had the highest allelic richness, which was 4.57. The five populations in Xishuangbanna, except that in Jinghong City, had the lowest allelic richness (3.55). The statistical test (one-way ANOWA) of the populations collected in 2019 and 2020 found that *p >* 0.05 (*p* = 0.086), indicating that the gene richness didn’t change significantly between 2 years.

**TABLE 4:** Allelic richness of all populations in 2019 and 2020.

Year	Region	Regional allelic richness	Population	No.	Population allelic richness
2019	Jinghong City	3.74	JH-1	30	3.84
			JH-2	30	4.15
			JH-3	30	3.42
			JH-4	30	3.49
			JH-5	30	4.11
			JH-6	30	3.79
			JH-7	30	3.44
			JH-8	30	3.73
			JH-9	30	3.66
	Other areas of Xishuangbanna	3.11	ML-1	30	3.44
			DL-1	30	3.36
			MH-1	30	2.52
	Ruili City	3.62	RL-1	30	3.54
			RL-2	30	3.48
			RL-3	30	3.84
	Lincang City	3.85	NS-1	30	3.35
			MD-1	30	4.18
			MD-2	30	3.74
			MD-3	30	4.11
			QSH-1	30	4.18
			QSH-2	30	3.53
	Myanmar	4.62	MD1	30	4.62
	Mean				3.71
2020	Jinghong City	3.88	JH-10	30	3.81
			JH-11	30	4.03
			JH-12	30	3.81
	Other areas of Xishuangbanna	3.55	ML-2	30	3.16
			ML-3	30	3.72
			DL-2	30	3.28
			DL-3	30	3.95
			MH-2	30	3.64
	Ruili City	4.57	RL-4	30	4.07
			RL-5	30	4.76
			RL-6	30	4.80
			RL-7	30	4.64
	Lincang City	3.95	NS-2	30	3.13
			MD-4	30	4.01
			MD-5	30	4.16
			MD-6	30	3.93
			QSH-3	30	4.51
	Mean				3.97

The number of alleles and allelic richness are both important indicators of population diversity. Comparing the 2-year sampling data, the average number of alleles obtained for the 22 populations in 2019 was 3.798, while the average number of alleles in 2020 was 4.353. The average allelic richness of the populations was 3.707 in 2019 and 3.965 in 2020. Regardless of the average number of alleles or the average allelic richness, the populations sampled in 2020 showed higher diversity than the populations sampled in 2019 (Table 3).

In this study, we used TPM and SMM models to analyze the bottleneck effect of samples collected in 2019 and 2020 (Table 5), and a total of six out of 22 natural populations sampled in 2019 showed a bottleneck effect. Three populations in Jinghong City (JH-1, JH-4, and JH-5), one population in Ruili City (RL-3), one population at Qingshuihe Port (QSH-2) and one population in Myanmar experienced a bottleneck effect. Among the 17 populations sampled in 2020, only the Jinghong City population (JH-11) and the Daluo Port (DL-2) population showed a bottleneck effect.

**TABLE 5:** Probability value for bottlenecks in 2019 and 2020.

Year	Population code	N	TPM	SMM
2019	JH-1	30	0.041*	0.392
	JH-2	30	0.604	0.607
	JH-3	30	0.135	0.380
	JH-4	30	0.042*	0.185
	JH-5	30	0.048*	0.552
	JH-6	30	0.600	0.555
	JH-7	30	0.371	0.598
	JH-8	30	0.387	0.582
	JH-9	30	0.600	0.548
	ML-1	30	0.632	0.335
	DL-1	30	0.350	0.571
	MH-1	30	0.064	0.080
	RL-1	30	0.120	0.621
	RL-2	30	0.527	0.205
	RL-3	30	0.028*	0.027*
	NS-1	30	0.623	0.349
	MD-1	30	0.371	0.337
	MD-2	30	0.339	0.622
	MD-3	30	0.604	0.330
	QSH-1	30	0.580	0.125
	QSH-2	30	0.010*	0.086
	MD1	30	0.560	0.025*
2020	JH-10	30	0.424	0.445
	JH-11	30	0.045*	0.405
	JH-12	30	0.616	0.617
	ML-2	30	0.214	0.063
	ML-3	30	0.399	0.113
	DL-2	30	0.040*	0.030*
	DL-3	30	0.285	0.186
	MH-2	30	0.155	0.421
	RL-4	30	0.358	0.129
	RL-5	30	0.615	0.139
	RL-6	30	0.628	0.135
	RL-7	30	0.325	0.120
	NS-2	30	0.396	0.154
	MD-4	30	0.623	0.337
	MD-5	30	0.615	0.147
	MD-6	30	0.623	0.598
	QSH-3	30	0.348	0.140

*** : *p* < 0.001; **: *p* < 0.01; *: *p* < 0.05.

### Microsatellite analysis of population structure and differences

**FIGURE 2:**
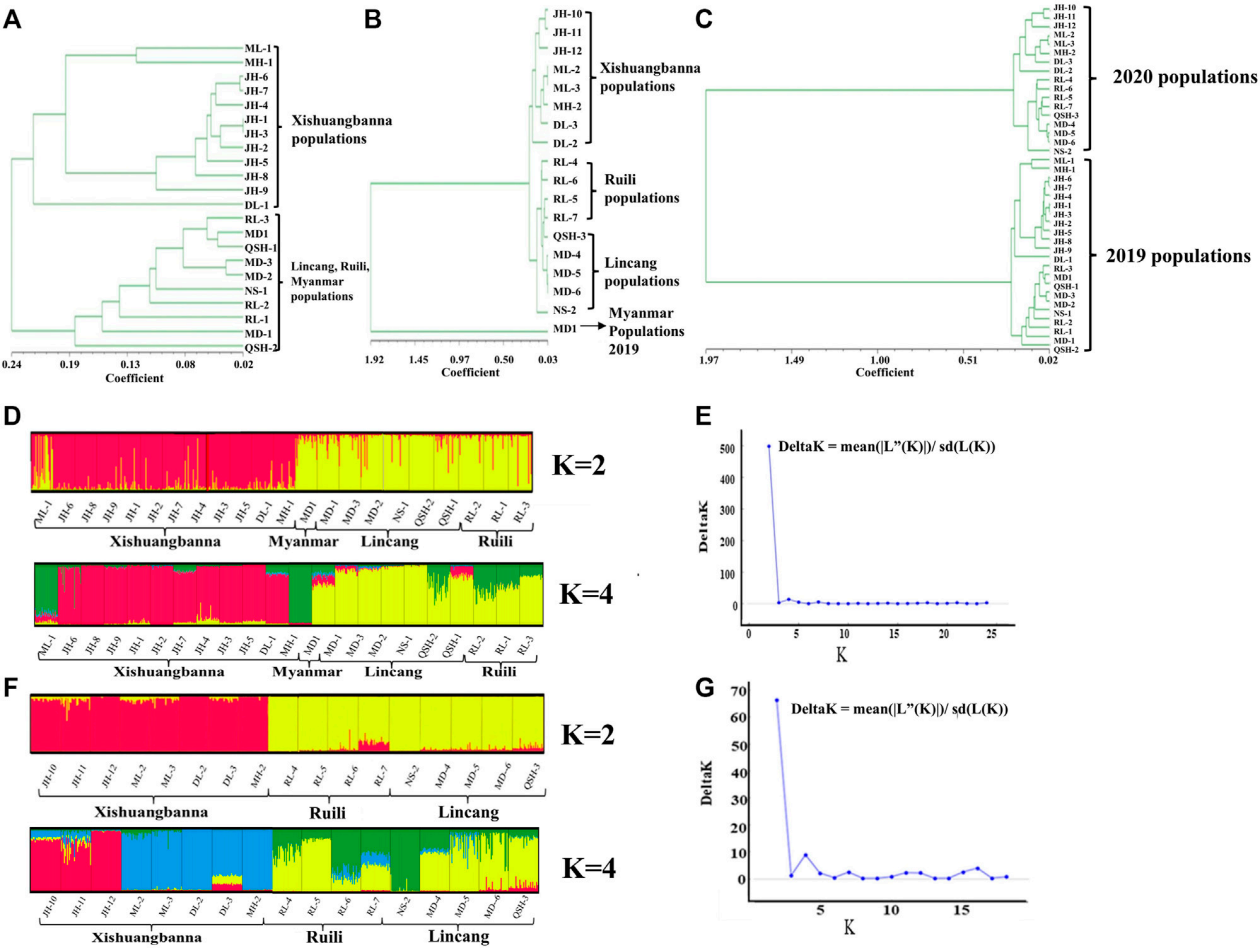
UPGMA **(A)**, **(B)**, **(C)** and STRUCTURE **(D)**, **(E)**, **(F)**, **(G)** cluster analysis. Figure 2A represents the UPGMA cluster analysis results of the populations collected in 2019; Figure 2B represents the UPGMA cluster analysis results of Myanmar population collected in 2019 and populations collected in the border area of Yunnan Province in 2020; Figure 2C the conjoint analysis of collected populations in 2019 and 2020; Figures 2D, F show the STRUCTURE cluster analysis of the populations collected in 2019 (K = 2, K = 4) and 2020 (K = 2, K = 4) respectively. The two black lines represent a population, and each color represents the percentage of individuals belonging to this cluster. The STRUCTURE software calculates that the optimal *k* value of the populations collected in 2019 and 2020 is 2 (represented by red and yellow bars respectively). Figures 2E, G represent K values of the populations collected in 2019 and 2020 assessed *via* Evanno et al.’s *ΔK* methods.

In this study, according to the microsatellite analysis results, both the UPGMA dendrogram and Evanno et al.’s *ΔK* methods (Evanno et al., 2005) divided the *Ae. aegypti* collected in Yunnan Province in 2019 into two clades (Figures 2A, D, E), with the 12 populations sampled in Xishuangbanna Prefecture grouped into one cluster and the nine populations sampled in Lincang City and Ruili City in Dehong Prefecture grouped into the other cluster. The Myanmar population was clustered with the populations from Lincang City and Ruili City. The population structure analysis results for the 17 populations sampled in 2020 were similar to those for the 2019 populations, which were also divided into two major clades. The eight populations sampled in Xishuangbanna Prefecture were clustered together, and the five populations sampled in Lincang City and four in Ruili City, Dehong Prefecture, were grouped together. The Myanmar population sampled in 2019 was separated from the populations collected in 2020 (Figures 2B, F, G). Based on the UPGMA method, we conducted a conjoint analysis of the populations in 2019 and 2020, found that 22 populations collected in 2019 were clustered into one group and 17 populations collected in 2020 were clustered into another group (Figure 2C).

**FIGURE 3:**
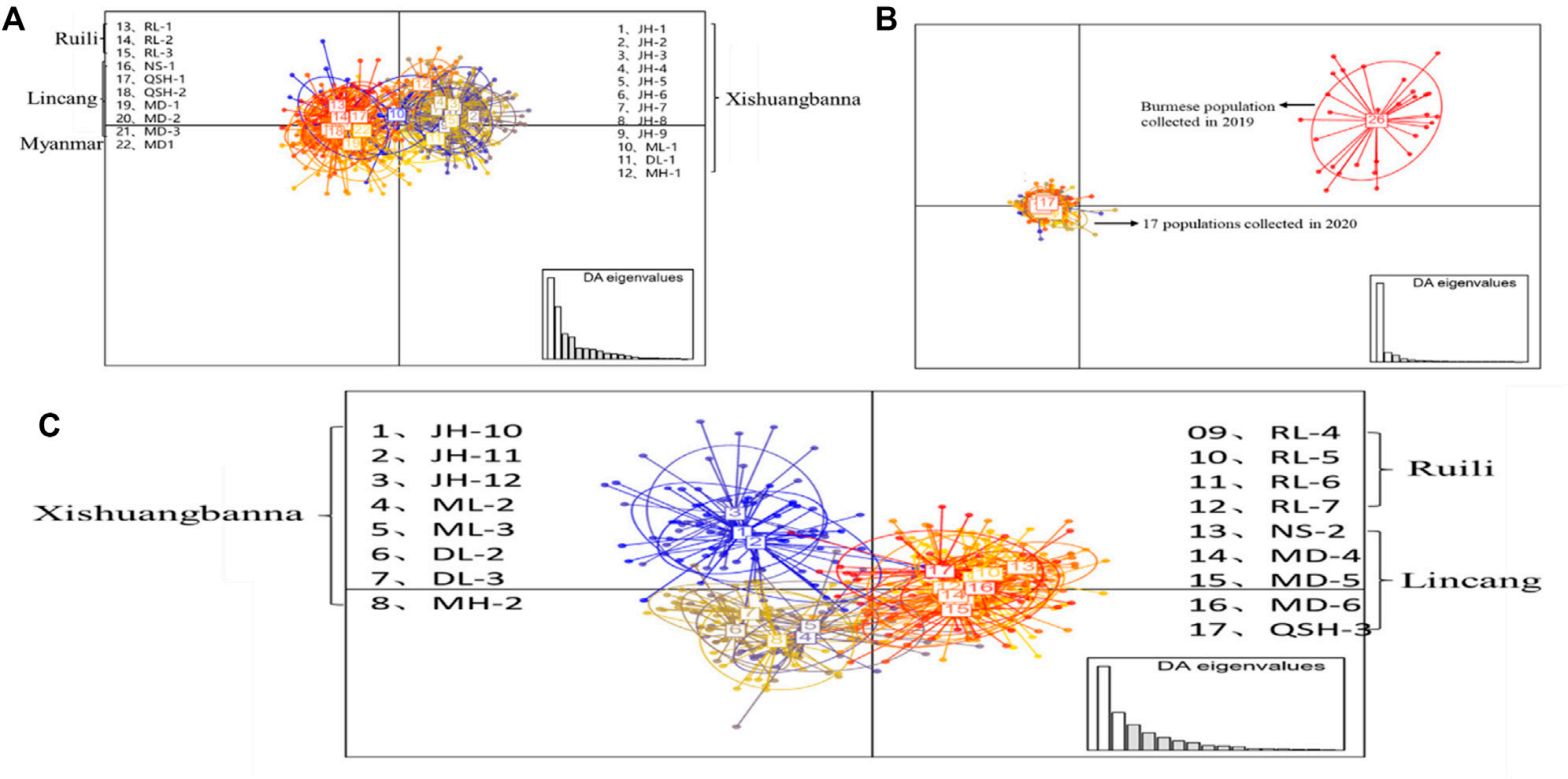
DAPC analysis of *Ae. aegypti* population structure based on microsatellite loci in the border areas of Yunnan Province. Each circle represents a cluster and each color represents the different subpopulations identified by the DAPC. Panel **(A)** represents the DAPC results for the 22 populations sampled in 2019; panel **(B)** represents DAPC results of collected population in 2019 and 2020; panel **(C)** represents the DAPC results for the 17 populations sampled in 2020.

The DAPC results for the 22 populations sampled in 2019 (Figure 3A) showed that the 12 populations in Xishuangbanna Prefecture (JH-1, JH-2, JH-3, JH-4, JH-5, JH-6, JH-7, JH-8, JH-9, ML-1, DL-1, MH-1) formed one cluster, while three populations (RL-1, RL-2, RL-3) in Ruili City, Dehong Prefecture, six populations (NS-1, QSH-1, QSH-2, MD-1, MD-2, MD-3) in Lincang City, and one population in Myanmar were clustered together. There was also some genetic communication between the two clusters. Figure 3B showed that the Myanmar population sampled in 2019 and the 17 populations sampled in 2020 were divided into two large groups, which didn’t overlap with each other. The results of DAPC of the 17 Yunnan populations sampled in 2020 are shown in Figure 3C, revealing that five populations (NS-2, MD-4, MD-5, MD-6, QSH-3) in Lincang City and four populations in Ruili City (RL-4, RL-5, RL-6, RL-7) were gathered into one cluster. The eight populations in Xishuangbanna Prefecture tended to intersect with each other. Overall, the clustering pattern was basically the same as that of the 2019 populations.

The AMOVA results (Table 6) showed that of the total genetic variation in the populations sampled in 2019, 88.18% could be attributed to differences within individuals and 8.40% to differences among populations within groups (Fit = 0.11822, Fsc = 0.09072, all *p* < 0.0001). Meanwhile, of the genetic variation in the populations sampled in 2020, 85.99% could be attributed to differences within individuals and 7.88% to differences among populations within groups (Fit = 0.14013, Fsc = 0.08433, all *p* < 0.0001).

**TABLE 6:** Results of AMOVA in 2019 and 2020.

Year	Source of variation	d.f	Sum of squares	Variance components	Percentage of variation	*p*-Value	Fixation index
2019	Among groups	1	149.240	0.20359 Va	7.44	*p* < 0.0001	F_CT_ = 0.07440
	Among populations within groups	20	319.607	0.22979 Vb	8.40	*p* < 0.0001	F_SC_ = 0.09072
	Among individuals within populations	638	1399.217	−0.10987 Vc	−4.02	*p* = 1.00000	F_IS_ = −0.04771
	Within individuals	660	1592.500	2.41288 Vd	88.18	*p* < 0.0001	F_IT_ = 0.11822
2020	Among groups	1	103.698	0.17468 Va	6.53	*p* < 0.0001	F_CT_ = 0.06533
	Among populations within groups	15	223.824	0.21073 Vb	7.88	*p* < 0.0001	F_SC_ = 0.08433
	Among individuals within populations	493	1122.817	−0.01075 Vc	−0.40	*p* > 0.05	F_IS_ = −0.00470
	Within individuals	510	1172.500	2.29902 Vd	85.99	*p* < 0.0001	F_IT_ = 0.14013

**FIGURE 4:**
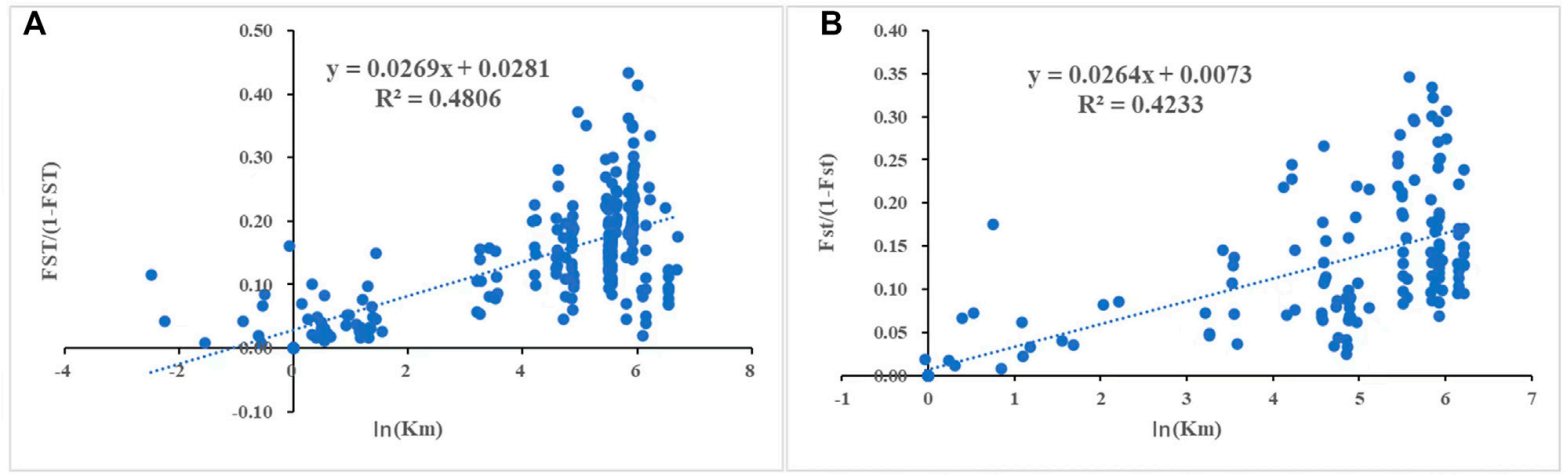
Analysis of the relationship between genetic distance [FST/(1-FST)] and geographical distance [㏑(km)] for **(A)** 22 populations sampled in 2019 (*R* ^2^ = 0.4806, *p* < 0.05) and **(B)** 17 populations sampled in 2020 (*R* ^2^ = 0.4233, *p* < 0.05)

The genetic distributions of 22 populations sampled in 2019 and 17 populations sampled in 2020 as obtained by the IBD model are shown in Figure 4 (Figure 4A 2019: *p* < 0.05 R = 0.6933; Figure 4B 2020: *p* < 0.05 R = 0.6506); the geographical distance and genetic distance of the populations were positively correlated in both years.

### Mitochondrial markers for the population structure and neutrality test

Based on COI gene analysis, 10 and 20 haplotypes were found in the populations sampled in 2019 and 2020, respectively.

Analyzing the ND4 fragments of the populations sampled in 2019 and 2020, we obtained a total of 17 haplotypes in the 2019 populations and 24 haplotypes in the 2020 populations.

The 30 haplotypes obtained for the COI gene of the populations sampled in the 2 years were compared and analyzed by MEGA software, revealing that the haplotypes H1, H2, H3, H4, H5, H6, H7, and H10 in 2019 were the same as H1, H7, H12, H2, H5, H20, H18, and H19 obtained from the 17 populations in 2020. Forty-one haplotypes of the ND4 gene were analyzed by the same method. Haplotypes H1, H2, H4, H5, H7, H10, H13, H15, and H17 obtained from the 22 populations in 2019 were the same as H1, H6, H15, H2, H19, H17, H20, H23, and H16 in the 2020 populations. GenBank IDs of COI haplotype are OM865371-OM865392. GenBank IDs of ND4 haplotype are OM877316-OM877347. Haplotype details were recorded in the Supplementary Appendix SA3.

**FIGURE 5:**
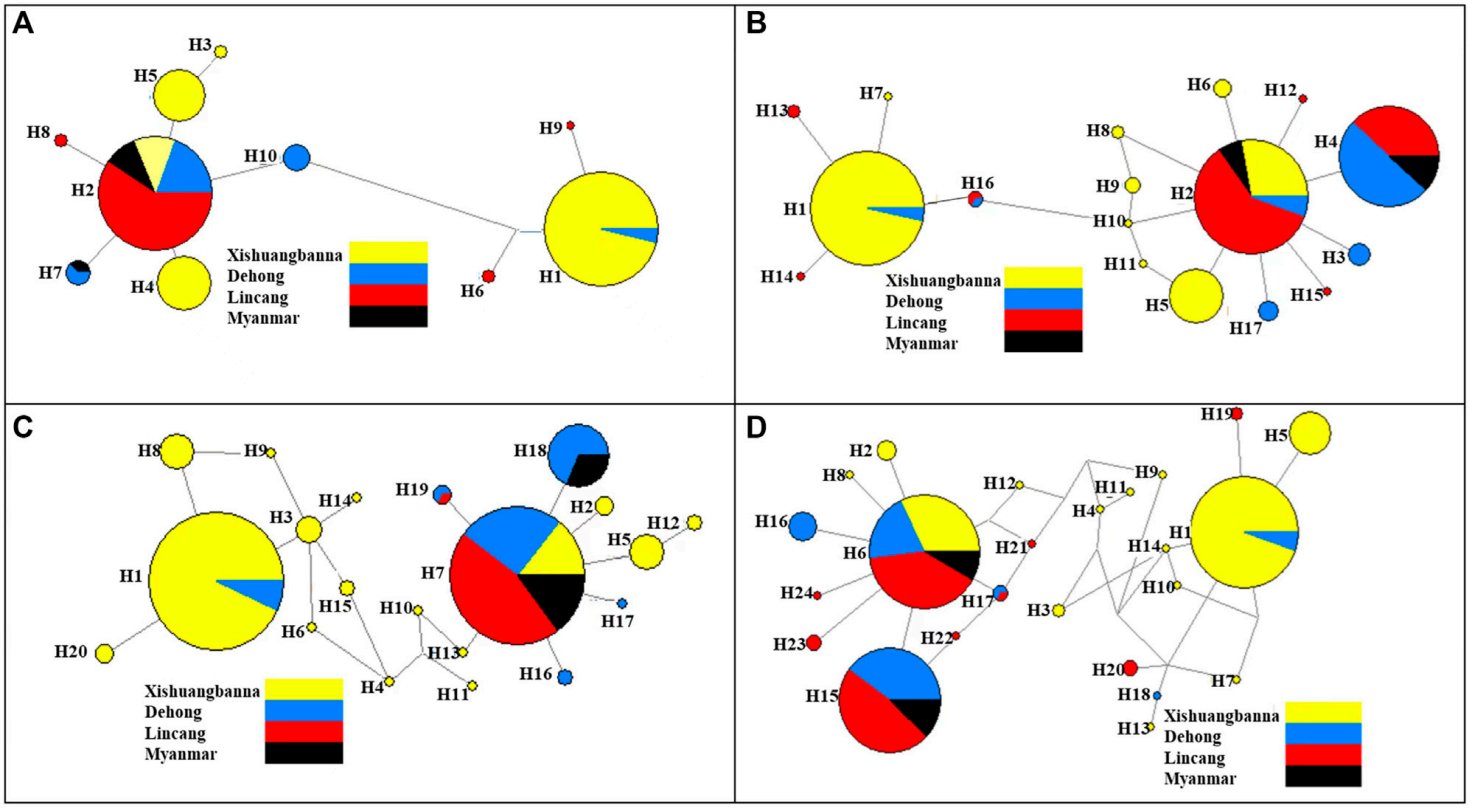
The haplotype network diagram based on COI and ND4 genes; **(A)** COI gene haplotype network diagram of populations in 2019; **(B)** ND4 gene haplotype network diagram of populations in 2019; **(C)** COI gene haplotype network diagram of populations in 2020; **(D)** ND4 gene haplotype network diagram of populations in 2020. The haplotypes of *Ae. aegypti* in each region are distinguished by different colors.

The haplotype network diagrams constructed with 653 COI fragments and 656 ND4 fragments obtained in 2019 showed that H1 and H2 were the most frequent haplotypes, and nearly all the other haplotypes derived were from H1 and H2 with several mutations. H1 haplotypes were mainly distributed in Xishuangbanna Prefecture, the other dominant haplotype H2 was mainly distributed in the Lincang City, Ruili City and Burmese populations. The COI and ND4 network diagram showed that the *Ae. aegypti* collected in 2019 were mainly divided into two clades, and the Myanmar population had more frequent exchanges with the populations of Lincang City and Ruili City (Figures 5A, B). The haplotype network diagram constructed based on the 508 COI fragments obtained in 2020 showed that H1 and H7 were two dominant haplotypes (Figures 5C, D). Analysis performed with MEGA software clearly showed that the H1 and H7 haplotypes of the COI fragments of the 2020 populations were consistent with the H1 and H2 haplotypes of the 2019 populations, respectively. The haplotype network diagram of 510 ND4 fragments showed that the two dominant haplotypes were H1 and H6, which were the same as the H1 and H2 haplotypes of the 2019 ND4 fragments (Additional files). The H1 haplotypes (COI and ND4 fragments) were mainly distributed in Xishuangbanna Prefecture, while the other dominant haplotypes H7 (COI fragments) and H6 (ND4 fragments) were mainly distributed in the Lincang City, Ruili City and Burmese populations sampled in 2019. In summary, the *Ae. aegypti* collected in 2020 were roughly divided into two clades, one in Xishuangbanna Prefecture and the other consisting of Lincang City and Ruili City populations sampled in 2020 and the Myanmar population sampled in 2019. This pattern was different from the clustering results based on microsatellite markers.

**FIGURE 6:**
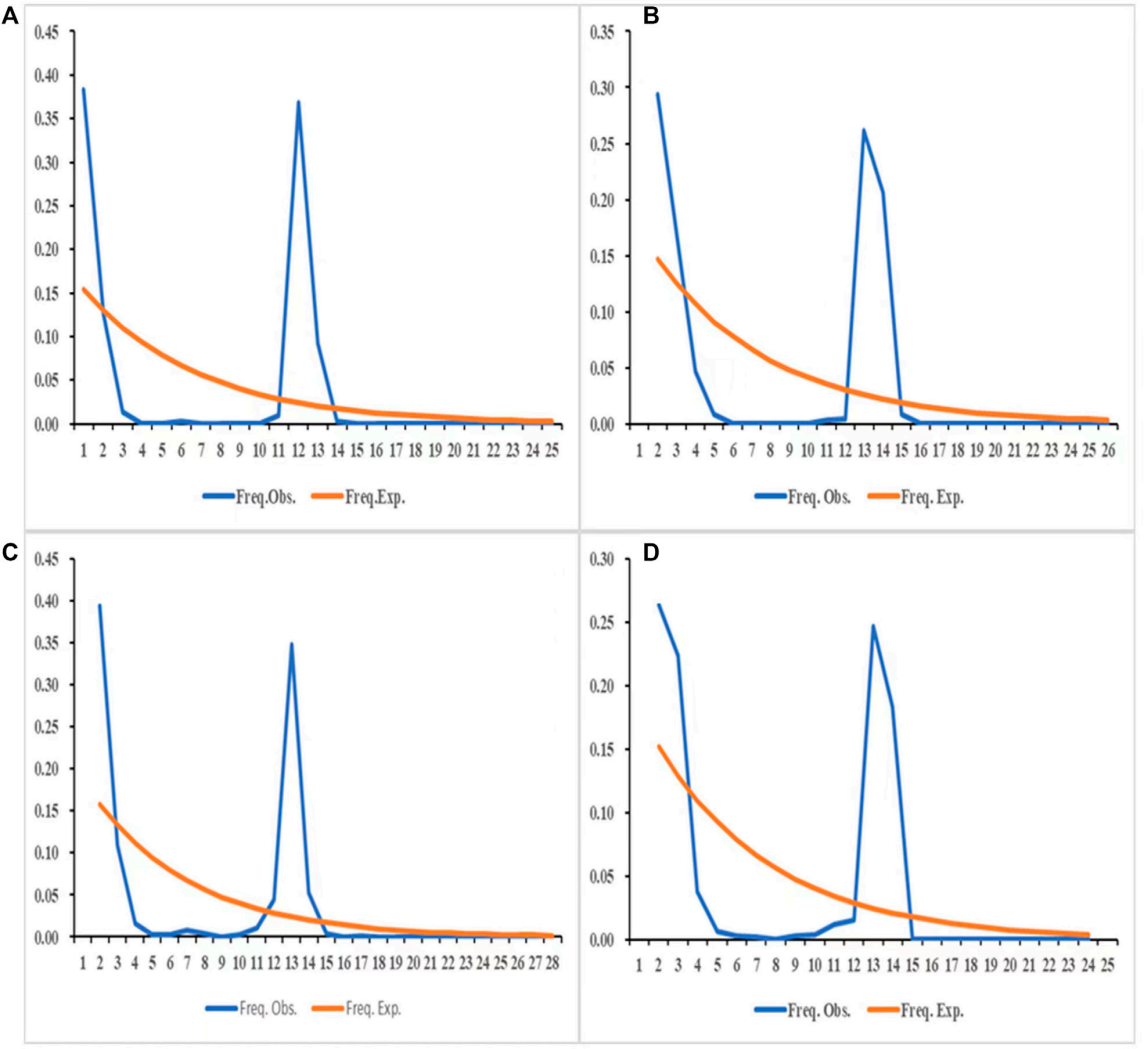
The distribution of the number of pairwise differences in COI and ND4 in 2019, 2020. **(A)** The distribution of the number of pairwise differences in COI in 2019. **(B)** The distribution of the number of pairwise differences in ND4 in 2019. **(C)** The distribution of the number of pairwise differences in COI in 2020. **(D)** The distribution of the number of pairwise differences in ND4 in 2020.

The results of the neutrality tests based on the COI and ND4 fragments of the sampled populations in 2019 (Table 7) showed that the Tajima’s D of JH-3 and JH-5 in Jinghong City, MD-3 in Mengding Town, QSH-1 and QSH-2 at Qingshuihe Port, and RL-2 in Ruili City were negative and had a *p* < 0.05, indicating that these populations had experienced bottleneck effects and then expanded. Based on the COI and ND4 fragments of the populations sampled in 2020 (Table 7), the neutrality test result showed that the JH-12 population in Jinghong City, the RL-7 population in Ruili City, and the MD-6 population in Mengding Town had historically experienced bottleneck effects. The nucleotide mismatch distribution (Figure 6) showed that the distribution curves of the populations in 2019 and 2020 had a single peak, indicating that *Ae. aegypti* underwent obvious population expansion in these areas.

**TABLE 7:** Neutrality test of 22 populations and 17 populations based on mtDNA in 2019, 2020.

Gene	Population	Tajima’s *D*	Tajima’s D *p-value*	Fu’s *FS*	FS *p-value*
COI (2019)	JH-1	0.379	>0.100	9.389	0.001
	JH-2	2.819	<0.010	11.217	0.000
	JH-3	−0.755	>0.100	6.918	0.009
	JH-4	0.000	1.000	0.000	N.A.
	JH-5	2.104	<0.050	7.790	0.002
	JH-6	2.948	<0.010	13.899	0.000
	JH-7	3.166	<0.001	11.738	0.000
	JH-8	1.195	>0.100	8.611	0.001
	JH-9	2.354	<0.050	12.925	0.000
	ML-1	2.604	<0.010	10.761	0.000
	DL-1	2.908	<0.010	13.053	0.000
	MH-1	0.000	1.000	0.000	N.A.
	RL-1	2.644	<0.010	12.642	0.000
	RL-2	0.880	>0.100	0.831	0.289
	RL-3	0.000	1.000	0.000	N.A.
	NS-1	0.000	1.000	0.000	N.A.
	QSH-1	−1.977	<0.050	1.867	0.176
	QSH-2	0.000	1.000	0.000	N.A.
	MD-1	0.000	1.000	0.000	N.A.
	MD-2	0.000	1.000	0.000	N.A.
	MD-3	−2.401	<0.010	2.726	0.195
	MD1	−0.409	>0.100	0.037	0.362
ND4 (2019)	JH-1	−0.172	>0.100	8.215	0.003
	JH-2	3.089	<0.001	10.931	0.000
	JH-3	−2.290	<0.010	7.817	0.002
	JH-4	0.000	1.000	0.000	N.A.
	JH-5	−2.351	<0.010	1.793	0.109
	JH-6	2.948	<0.010	13.899	0.000
	JH-7	3.193	<0.001	11.080	0.000
	JH-8	1.479	>0.100	8.496	0.002
	JH-9	2.321	<0.050	12.128	0.000
	ML-1	3.347	<0.001	13.723	0.000
	DL-1	2.908	<0.010	13.053	0.000
	MH-1	0.000	1.000	0.000	N.A.
	RL-1	0.806	>0.100	9.497	0.001
	RL-2	−2.781	<0.001	16.280	0.000
	RL-3	0.733	>0.100	3.346	0.134
	NS-1	0.000	1.000	0.000	N.A.
	QSH-1	−1.820	<0.050	0.894	0.215
	QSH-2	−1.883	<0.050	4.867	0.030
	MD-1	0.727	>0.100	1.080	0.372
	MD-2	−0.764	>0.100	−0.439	0.310
	MD-3	−2.153	<0.050	0.202	0.225
	MD1	1.621	>0.100	1.700	0.315
COI (2019)	JH-10	−0.579	>0.100	3.283	0.077
	JH-11	0.499	>0.100	6.877	0.006
	JH-12	−2.206	<0.010	0.359	0.252
	ML-2	1.687	>0.100	6.351	0.006
	ML-3	0.474	>0.100	1.386	0.756
	DL-2	−1.147	>0.100	−1.211	0.204
	DL-3	2.113	<0.050	7.240	0.004
	MH-2	−0.528	>0.100	−0.487	0.234
	RL-4	2.324	<0.050	9.805	0.000
	RL-5	−1.256	>0.100	−1.669	0.128
	RL-6	−0.750	>0.100	0.809	0.289
	RL-7	−2.087	<0.050	0.627	0.250
	NS-2	0.000	1.000	NA	NA
	MD-4	0.000	1.000	NA	NA
	MD-5	0.000	1.000	NA	NA
	MD-6	−2.401	<0.010	2.726	0.195
	QSH-3	−1.537	>0.100	3.067	0.110
ND4 (2019)	JH-10	−0.524	>0.100	3.394	0.071
	JH-11	0.445	>0.100	8.864	0.002
	JH-12	−2.121	<0.050	1.801	0.219
	ML-2	2.157	<0.050	7.866	0.002
	ML-3	0.874	>0.100	1.452	0.156
	DL-2	0.000	1.000	NA	NA
	DL-3	3.147	<0.001	7.961	0.002
	MH-2	0.000	1.000	NA	NA
	RL-4	2.618	<0.010	6.072	0.008
	RL-5	0.489	>0.100	0.519	0.295
	RL-6	0.604	>0.100	0.283	0.252
	RL-7	−1.911	<0.05	0.995	0.237
	NS-2	0.000	1.000	NA	NA
	MD-4	−0.048	>0.100	−1.405	0.126
	MD-5	−0.409	>0.100	0.037	0.362
	MD-6	−1.918	<0.050	2.243	0.177
	QSH-3	−1.292	>0.100	1.586	0.175

## Discussion

Molecular markers were previously used to analyze the genetic characteristics of *Ae. aegypti* populations in the border areas of Yunnan Province (Shi et al., 2017b). However, very few studies have focused on the changes in the genetic characteristics of *Ae. aegypti* populations under different selection pressures. *Ae. aegypti* in the border areas of Yunnan were collected in 2019 and 2020, respectively, and this study focused on analyzing the changes in the genetic characteristics of *Ae. aegypti* populations under different immigration pressures, especially the outbreak of dengue that occurred in 2019, where an integrated vector management approach was implemented. Because of the COVID-19 pandemic, the border was closed and crossings were limited in 2020, which led to declines in person and logistic flows. The status of the populations over 2 years provides an opportunity to study the population structure of *Ae. aegypti* with prevention and control pressures and human intervention related to COVID-19, thereby revealing the impact of human intervention, which will have important guiding significance for the prevention and control of this population in the future.

### The genetic diversity of *Ae. Aegypti* populations in border areas of Yunnan Province

Null alleles are one of the biggest defects of microsatellite markers; they mainly reduce population genetic diversity and increase genetic differentiation between populations (Chapuis and Estoup, 2007; Girard, 2011; Rico et al., 2017). Yan et al. found that the number of alleles increases with increasing sample size. A minimum sample size of 30–50 individuals is required for microsatellite DNA analysis (Luna and Dexing, 2004). The sample sizes collected in this study in 2019 and 2020 were both 30, and this sample size can reflect the true diversity of the population. The PIC of nine loci in 22 populations sampled in 2019 reached 0.546, and the PIC of 17 populations sampled in 2020 was 0.545; both values exceeded the standard for inferring high polymorphism. The diversity index results showed that a total of 67 alleles were obtained from 22 populations in 2019, and a total of 72 alleles were obtained from 17 populations in 2020. The Shannon index (SI) is another important factor reflecting population diversity. The average SI of the sampled populations in 2019 was 0.971. The average SI of the sampled populations in 2020 was 0.969. Thus, it could be concluded that the genetic diversity of the 17 populations in 2020 had little change compared with that of the 22 populations in 2019. The average observed heterozygosity of the populations sampled in 2019 was 0.538, while the average expected heterozygosity was 0.517, indicating that the *Ae. aegypti* populations sampled in 2019 had immigration. Considering that the flight distance of *Ae. aegypti* is usually short, it is possible that the immigrants mainly came from the flow of people or goods. In 2020, the average observed heterozygosity of the sampled populations was 0.514, and the average expected heterozygosity was 0.519, which were basically the same, indicating that the populations experienced inbreeding. This may be due to the outbreak of COVID-19 in early 2020. China quickly took strong measures to control the epidemic. However, the epidemic prevention abroad was not optimistic. To reduce the risk of foreign imports, the management of the movement of people and goods was strengthened at Chinese ports. As a result, the opportunity for invasion and number of invading individuals of *Ae. aegypti* from neighboring countries through the movement of people and goods were greatly reduced.

The diversity index of the populations sampled in 2019 and 2020 was relatively high after microsatellite marker analysis. In summary, the overall diversity of *Ae. aegypti* populations in Yunnan Province was relatively high. Stable populations have been established and have the ability to resist adverse external environmental influences and expansion. The flow restriction of people and goods had a certain impact on the populations of *Ae. aegypti*. If the populations cannot be fully supplemented in the long run, the diversity of the *Ae. aegypti* in border areas of Yunnan Province may decline.

### The current colonization and expansion patterns of *Ae. aegypti* in border areas of Yunnan Province

Generally, only a few individuals of invasive species can enter new areas, and newly invasive individuals will experience genetic bottleneck effects after arriving in the new areas, which will eventually lead to a decrease in genetic diversity (Tabachnick et al., 1979). The bottleneck effect analysis based on the microsatellite markers of the populations sampled in 2019 revealed that a total of six natural populations (JH-1, JH-4, JH-5, RL-3, QSH-2, MD1) sampled in 2019 experienced a bottleneck effect. Among them, the three populations in Jinghong City had positive inbreeding coefficients (Fis), indicating inbreeding in the populations. The bottleneck effect may be due to the serious dengue fever epidemic in Jinghong City and subsequent focus on vector control. The QSH-2 population at Qingshuihe Port and the RL-3 population in Ruili City both came from ports, with low genetic diversity and a negative inbreeding coefficient (Fis). Therefore, it was speculated that these two populations were more likely to be new invasively colonized. Two of the populations (JH-11 and DL-2) sampled in 2020 experienced a bottleneck effect. The collection locations of these two populations were far from the ports, and the inbreeding coefficient (Fis) was positive, so it was inferred that the local control of mosquito vectors caused the bottleneck effect. The neutrality test results based on the COI and ND4 fragments showed that six of the populations sampled in 2019 (JH-3, JH-5, MD-3, QSH-1, QSH-2, RL-2) experienced a bottleneck effect, and the 2020 populations JH-12, RL-7, MD-6 also experienced bottleneck effects. If the distribution of the number of pairwise differences shows a single peak and followed a Poisson distribution, it indicates that the population experienced expansion recently, whereas multiple peaks indicates that the population was in a stable state recently (Harpending, 1994). The nucleotide mismatch distribution analysis performed in this study showed that the distribution curves had a single peak, indicating that *Ae. aegypti*, as an invasive mosquito species, experienced population expansion in Yunnan Province in 2019 and 2020.

In the port areas of Yunnan Province, the bottleneck effect impacting the populations occurred in the 2 years, indicating continuous invasion of *Ae. aegypti* in the port areas. The bottleneck effect experienced by the populations in Jinghong City may be due to the outbreak of dengue fever year after year, and the *Ae. aegypti* populations face great environmental pressure, such as disinfection and sterilization, so it is difficult for them to maintain a stable colonization state. The populations in other regions have not experienced bottleneck effects and were in a stable state. The nucleotide mismatch distribution analysis of the populations sampled in 2019 and 2020 revealed that *Ae. aegypti* in Yunnan Province had experienced expansion. This may be because although the populations sampled in 2020 received less gene flow, the population diversity was similar to that of the 2019 populations, and it also had the ability to expand.

### The population genetic characteristics of *Ae. aegypti* under different immigration pressures

The cluster analysis results of the populations sampled in 2019 based on microsatellite markers and mitochondrial DNA showed that the populations can be divided into two major clades; one clade included the populations of *Ae. aegypti* in Xishuangbanna Prefecture, and the other clade included the populations of Lincang City, Ruili City, Dehong Prefecture and Myanmar. The Myanmar population had the highest allelic richness. Generally, multiple invasions can increase the genetic diversity of the invading population, but the overall level will not be higher than that of the source populations. With spread, genetic diversity will further decline (Dlugosch and Parker, 2010; Uller and Leimu, 2011); therefore, the population of *Ae. aegypti* in Myanmar is likely a source population of *Ae. aegypti* in Yunnan Province. Comparing the allelic richness of various populations in Yunnan Province sampled in 2019, the Lincang City populations had higher values than the Ruili City populations. Because the Lincang City and Ruili City populations were located in the border areas, the Lincang populations likely didn’t directly spread from the Ruili populations but invaded from Myanmar, which indicates that invasion followed not a single route but multiple routes. Jinghong City is far from the border, the Lancang River flows through the city, tourism is developed, and freight transport is frequent. Therefore, the populations of *Ae. aegypti* in Jinghong City may come from invasion by foreign populations with travelers and then spread to other areas of Xishuangbanna Prefecture. They may also come from Laos bordering Mohan Port and eastern Myanmar bordering Daluo Port.

Microsatellite molecular marker analysis showed that the Burmese population sampled in 2019 and the Yunnan populations sampled in 2020 could not be grouped together. However, the haplotype network map constructed by mitochondrial DNA molecular markers revealed that the Myanmar population sampled in 2019 and the populations in Lincang and Ruili sampled in 2020 shared haplotypes. The dominant haplotypes didn’t change between the 2 years. This phenomenon may be caused by the fact that the mutation rate of mitochondrial DNA molecular markers is lower than that of microsatellite markers. In summary, the *Ae. aegypti* populations in the border areas of Yunnan Province in 2020 may have come from a new invasion of Myanmar, or they may have resulted from breeding of the *Ae. aegypti* population in Yunnan Province after natural selection and vector control in 2019. There was inbreeding in 17 populations sampled in Yunnan Province in 2020. Considering the situation at the border ports, due to the impact of COVID-19, the flow of people and goods was reduced, and the disinfection and sterilization of goods entering Yunnan through ports were more effective than before. As a result, the probability of Burmese *Ae. aegypti* population invasion with the flow of people and logistics decreased.

## Conclusion

The genetic diversity of *Ae. aegypti* in Yunnan Province was relatively high, and the populations were mainly divided into two major clusters. The main reason for the formation of the two clusters may be different invasion events. The continued invasion of *Ae. aegypti* in border areas maintained population diversity. Under different immigration pressures, prevention and control pressures, the structure and diversity of the *Ae. aegypti* populations could change. Human activities may be one cause of the expansion of *Ae. aegypti* in the border areas of Yunnan Province. These research results can provide some guidance for the prevention and control of *Ae. aegypti* in China.

## Data Availability

The datasets presented in this study can be found in online repositories. The names of the repository/repositories and accession number(s) can be found in the article/Supplementary Material.
