# Supplementary material for: Population genetic characteristics of Aedes aegypti in 2019 and 2020 under the distinct circumstances of dengue outbreak and the COVID-19 pandemic in Yunnan Province, China
Source: Front Genet. 2023 Mar 9;14:1107893. doi: 10.3389/fgene.2023.1107893 (PMC10033842; doi:10.3389/fgene.2023.1107893)
Supplement: Supplementary file 1 [file DataSheet1.ZIP › Appendix/Appendix 1. Aedes aegypti microsatellite primer information/Appendix 1. Aedes aegypti microsatellite primer information.docx]

Appendix 1.  *Ae. aegypti* microsatellite primer information

| Locus | Repeat unit | Sequence 5’to3’ | Annealing temperature | Length | Label |
| --- | --- | --- | --- | --- | --- |
| SQM1 | CT10（TT）CT | F: AATCGTGACGCGTCTTTTG | 54℃ | 233~239 bp | 5'-HEX |
|  |  | R: TAACTGCATCGAGGGAAACC |  |  |  |
| SQM2 | GA15 | F: CAAACAACGAACTGCTCACG | 53℃ | 157~183 bp | 5'-FAM |
|  |  | R: TCGCAATTTCAACAGGTAGG |  |  |  |
| SQM3 | CAT7 | F: ATTGGCGTGAGAACATTTTG | 58℃ | 156~186 bp | 5'-FAM |
|  |  | R: GAGGAGTGAGCAGATAGGAGTG |  |  |  |
| SQM4 | TAGA8 | F: GCCAAAAACCAACAAACAGG | 53℃ | 286~290 bp | 5'-TAMRA |
|  |  | R: AATCGACCCGACCAATAACA |  |  |  |
| SQM5 | ATA36 | F: GGAGCATTCATAGAGAATTGTCA | 56℃ | 110~116 bp | 5'-FAM |
|  |  | R: GAGATGAACCAGTCATAGGGC |  |  |  |
| SQM6 | （TTTA）7（T）14 | F: CGACAGATGGTTACGGACGG | 58℃ | 228 bp | 5'-FAM |
|  |  | R: GTCCCGCTCCAAAAATGCCC |  |  |  |
| SQM7 | AG4 | F: AAAACCTGCGCAACAATCAT | 56℃ | 147~169 bp | 5'-FAM |
|  |  | R: AAGGACTCCGTATAATCGCAAC |  |  |  |
| SQM8 | AG5 | F: TGATCTTGAGAAGGCATCCA | 55℃ | 170~180 bp | 5'-FAM |
|  |  | R: CGTTATCCTTTCATCACTTGTTTG |  |  |  |
| SQM9 | AC1 | F: TCCGGTGGGTTAAGGATAGA | 55℃ | 193~209 bp | 5'-FAM |
|  |  | R: ACTTCACGCTCCAGCAATCT |  |  |  |

SQM is the naming of locus in the article I quoted.
