# Supplementary figures and images for: Population genetic characteristics of Aedes aegypti in 2019 and 2020 under the distinct circumstances of dengue outbreak and the COVID-19 pandemic in Yunnan Province, China
Source: Front Genet. 2023 Mar 9;14:1107893. doi: 10.3389/fgene.2023.1107893 (PMC10033842; doi:10.3389/fgene.2023.1107893)

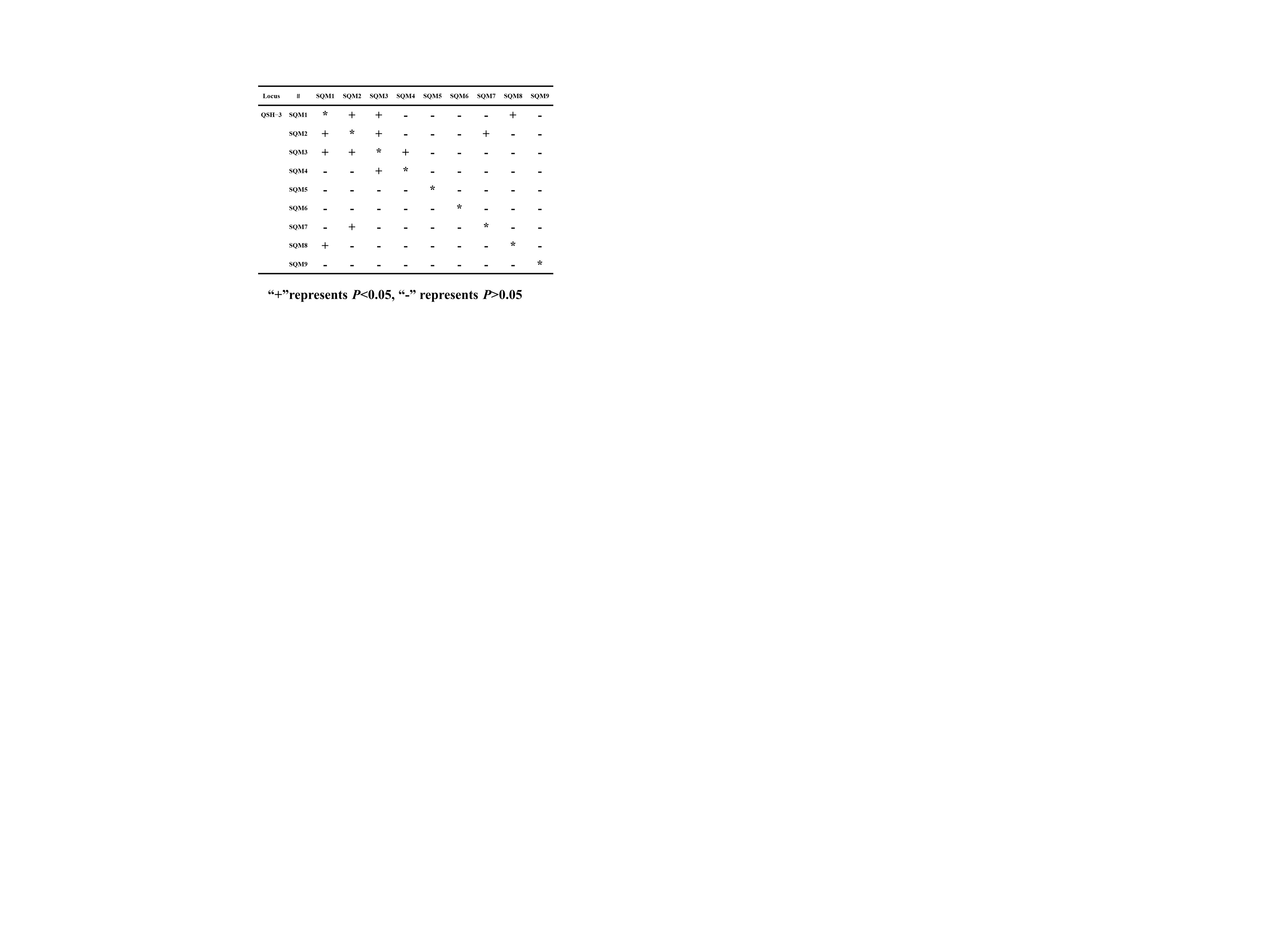

Supplement: Supplementary file 1 [file DataSheet1.ZIP › Appendix/Appendix 2. Analysis of linkage disequilibrium in 17 populations/Appendix 3. Analysis of linkage disequilibrium in 17 populations/Appendix 3. Analysis of linkage disequilibrium in 17 populations ú¿3ú⌐.jpg]

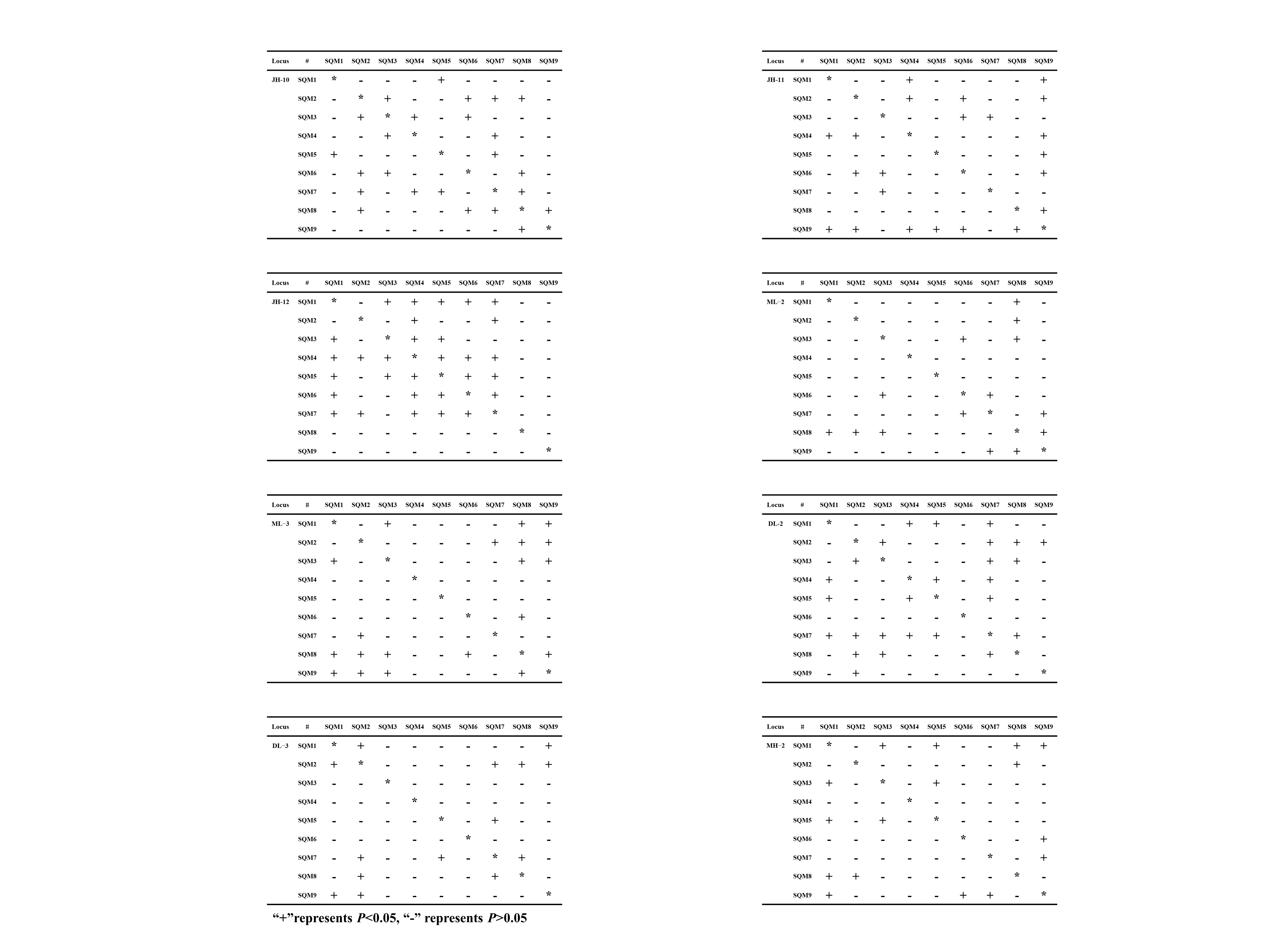

Supplement: Supplementary file 1 [file DataSheet1.ZIP › Appendix/Appendix 2. Analysis of linkage disequilibrium in 17 populations/Appendix 3. Analysis of linkage disequilibrium in 17 populations/Appendix 3. Analysis of linkage disequilibrium in 17 populationsú¿1ú⌐.jpg]

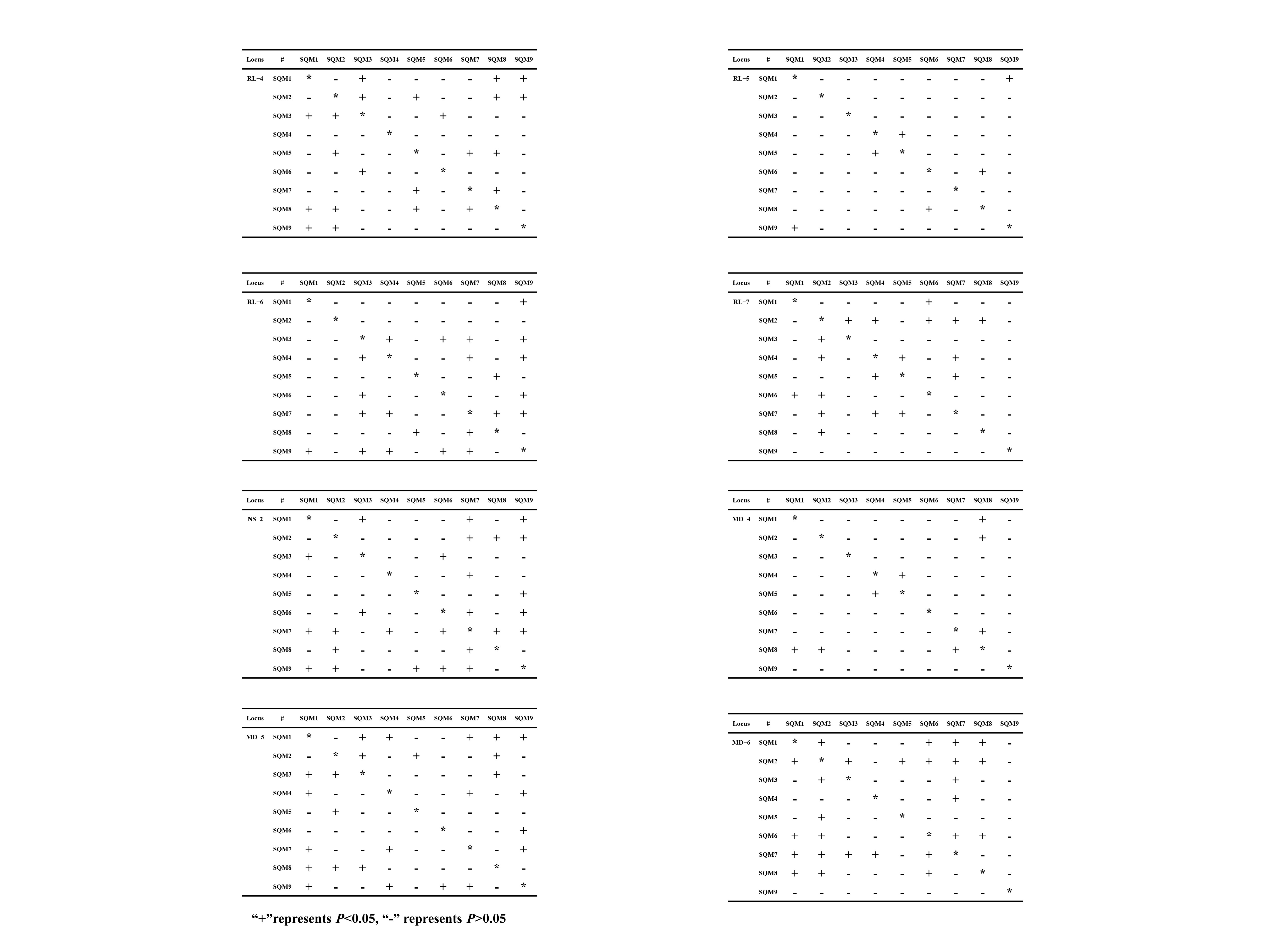

Supplement: Supplementary file 1 [file DataSheet1.ZIP › Appendix/Appendix 2. Analysis of linkage disequilibrium in 17 populations/Appendix 3. Analysis of linkage disequilibrium in 17 populations/Appendix 3. Analysis of linkage disequilibrium in 17 populationsú¿2ú⌐.jpg]

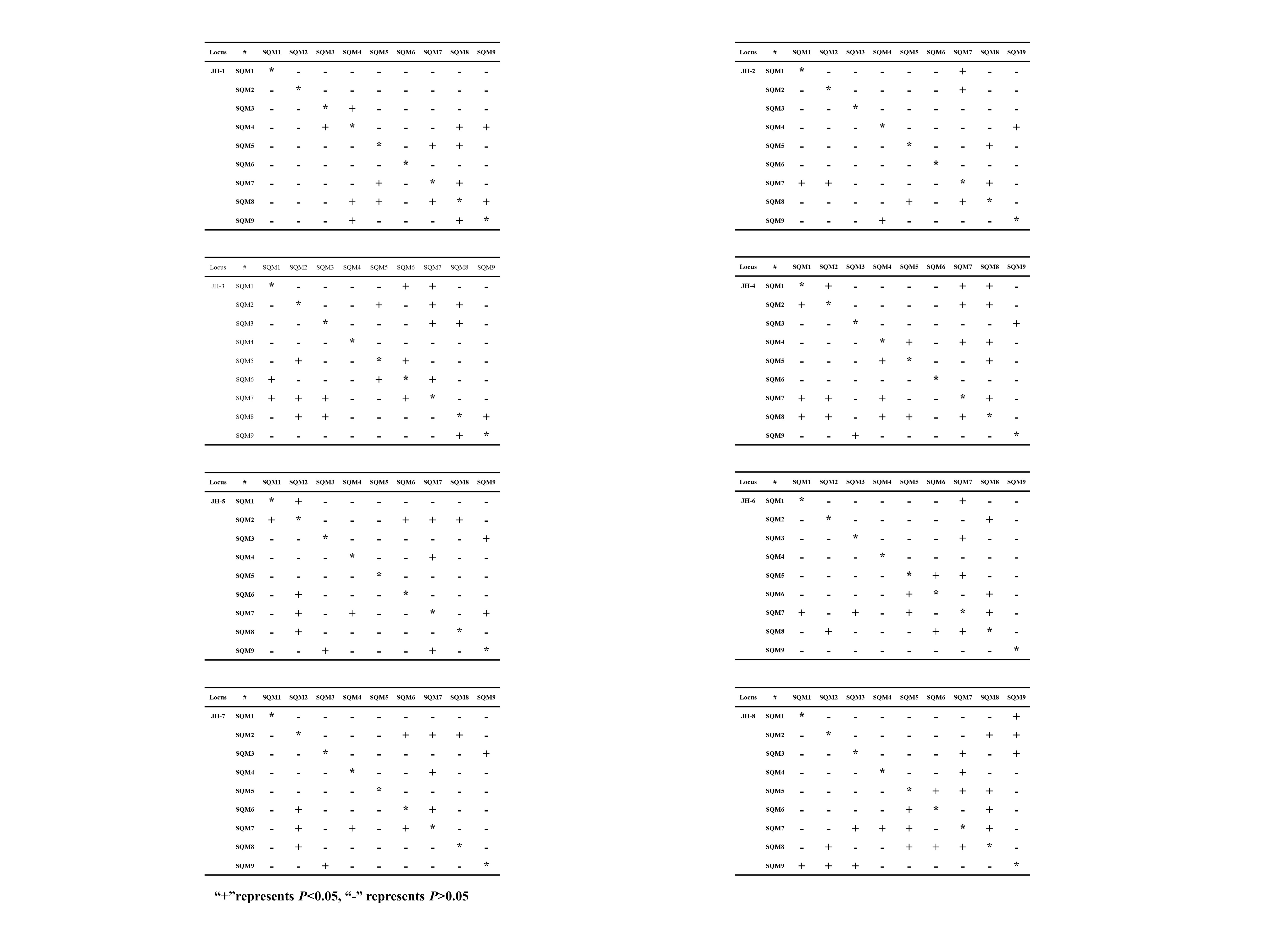

Supplement: Supplementary file 1 [file DataSheet1.ZIP › Appendix/Appendix 2. Analysis of linkage disequilibrium in 22 populations/Appendix 3. Analysis of linkage disequilibrium in 22 populations/Appendix 3. Analysis of linkage disequilibrium in 22 populations ú¿1ú⌐.jpg]

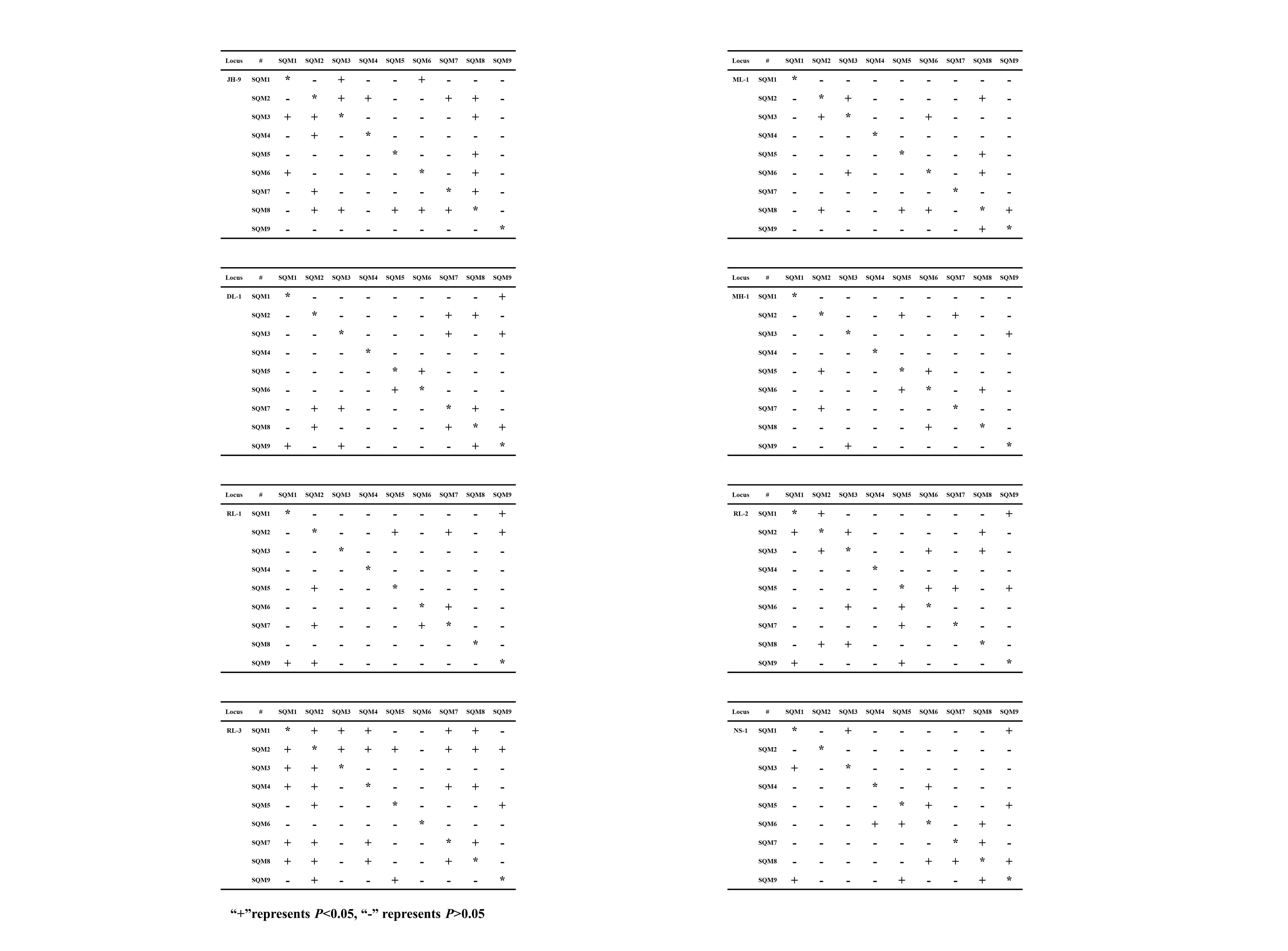

Supplement: Supplementary file 1 [file DataSheet1.ZIP › Appendix/Appendix 2. Analysis of linkage disequilibrium in 22 populations/Appendix 3. Analysis of linkage disequilibrium in 22 populations/Appendix 3. Analysis of linkage disequilibrium in 22 populations ú¿2ú⌐.jpg]

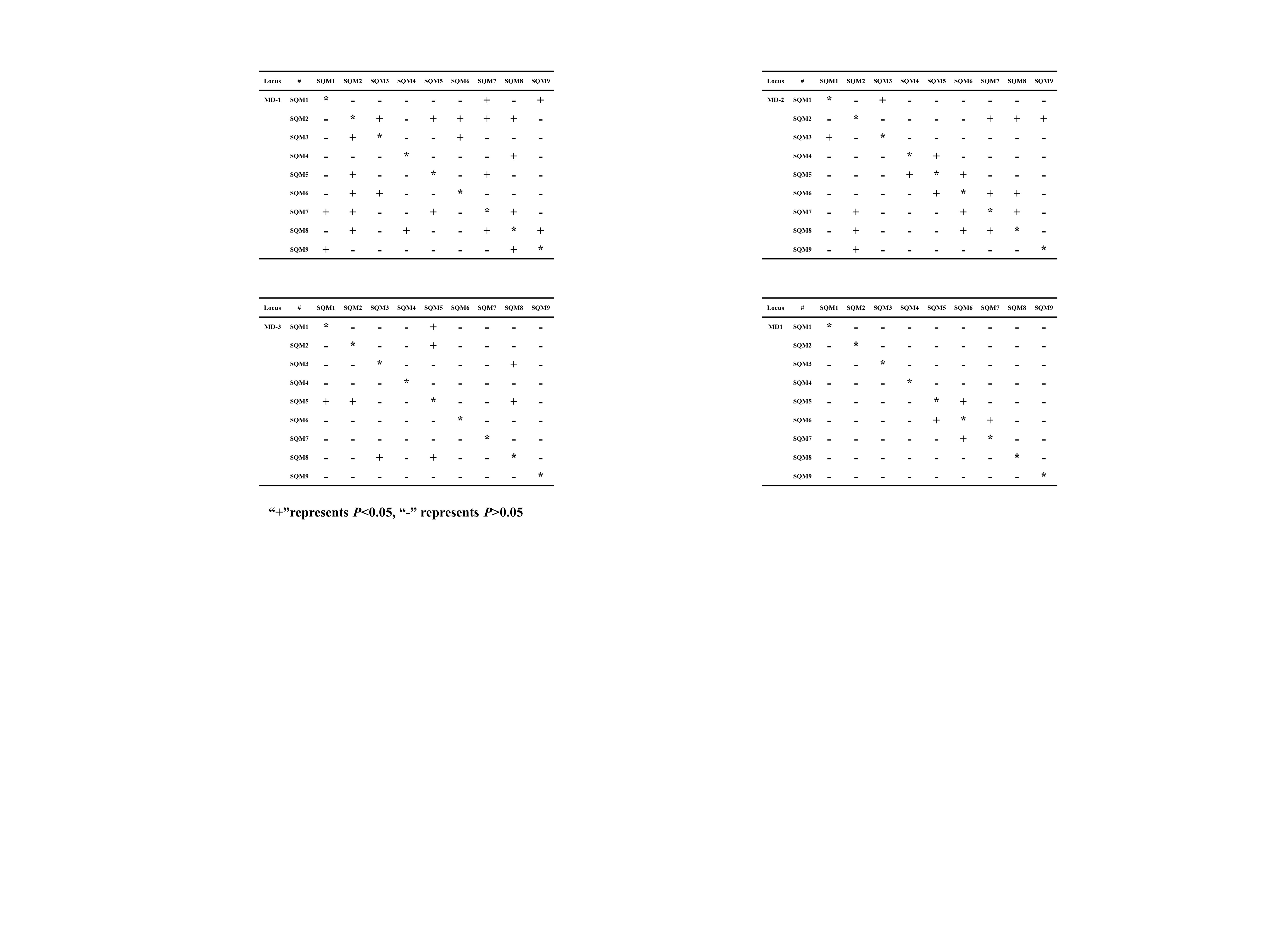

Supplement: Supplementary file 1 [file DataSheet1.ZIP › Appendix/Appendix 2. Analysis of linkage disequilibrium in 22 populations/Appendix 3. Analysis of linkage disequilibrium in 22 populations/Appendix 3. Analysis of linkage disequilibrium in 22 populations ú¿3ú⌐.jpg]

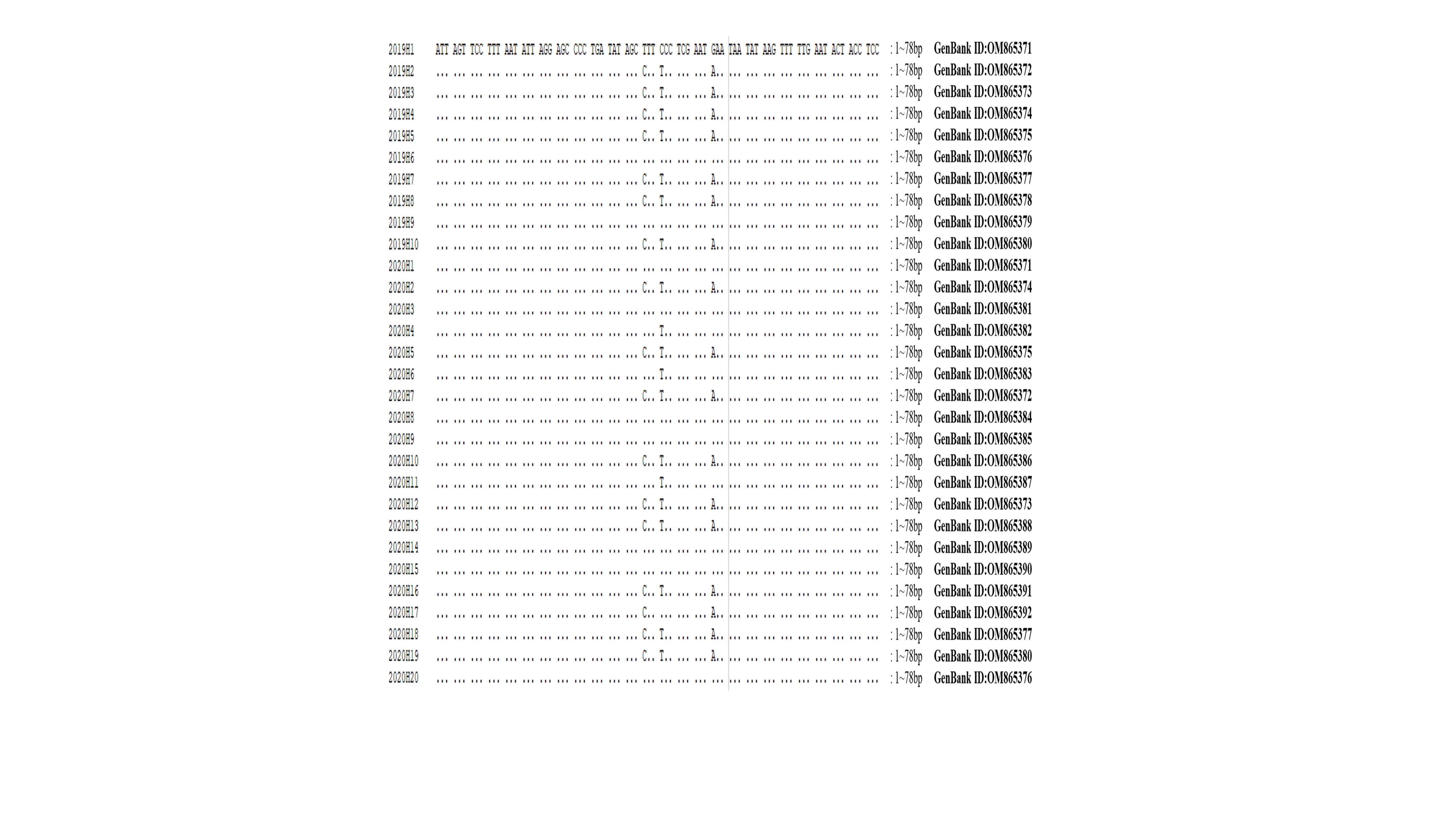

Supplement: Supplementary file 1 [file DataSheet1.ZIP › Appendix/Appendix 3. Haplotype information based on the mitochondrial COó± gene of Ae. aegypti/1.jpg]

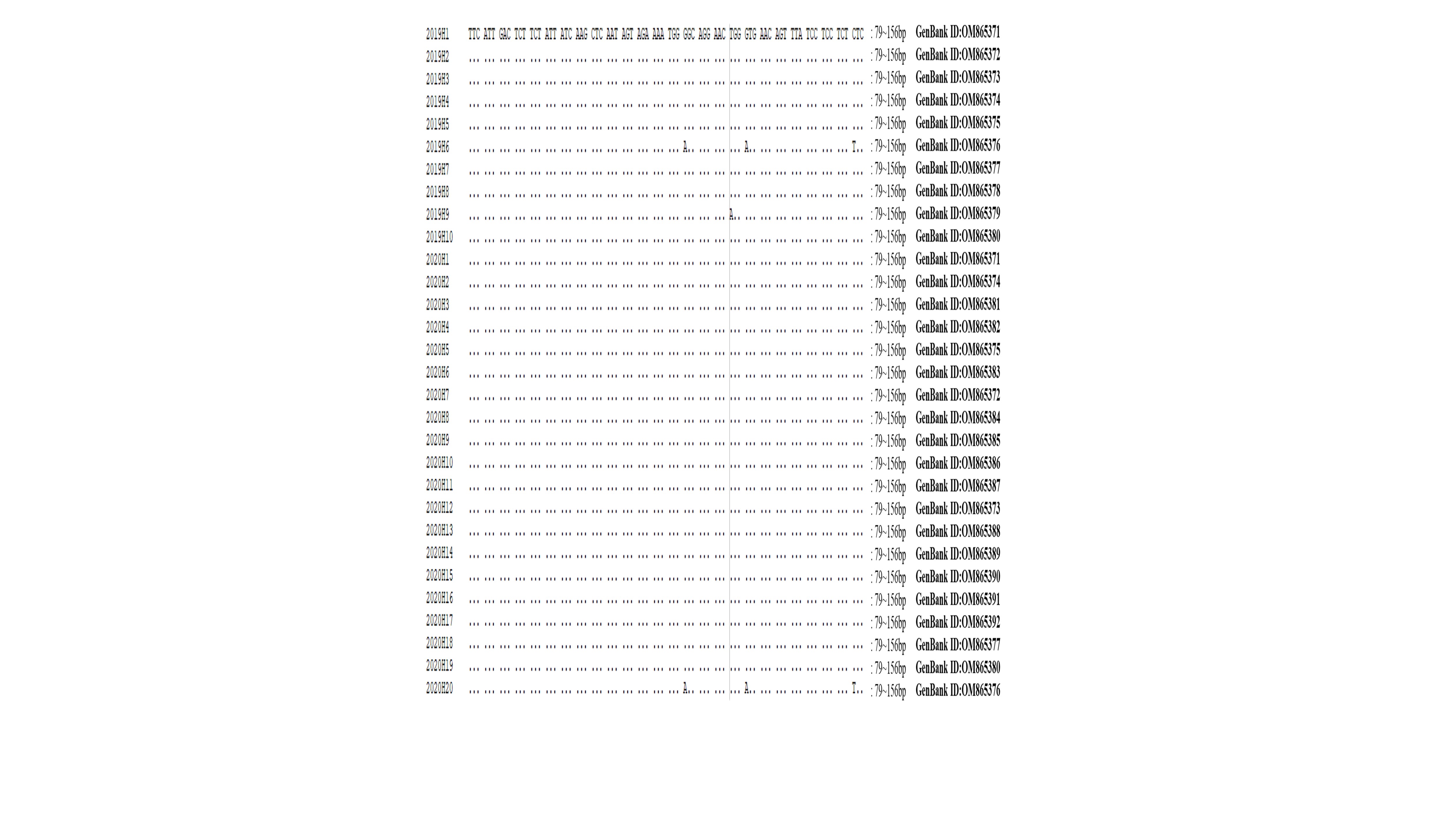

Supplement: Supplementary file 1 [file DataSheet1.ZIP › Appendix/Appendix 3. Haplotype information based on the mitochondrial COó± gene of Ae. aegypti/2.jpg]

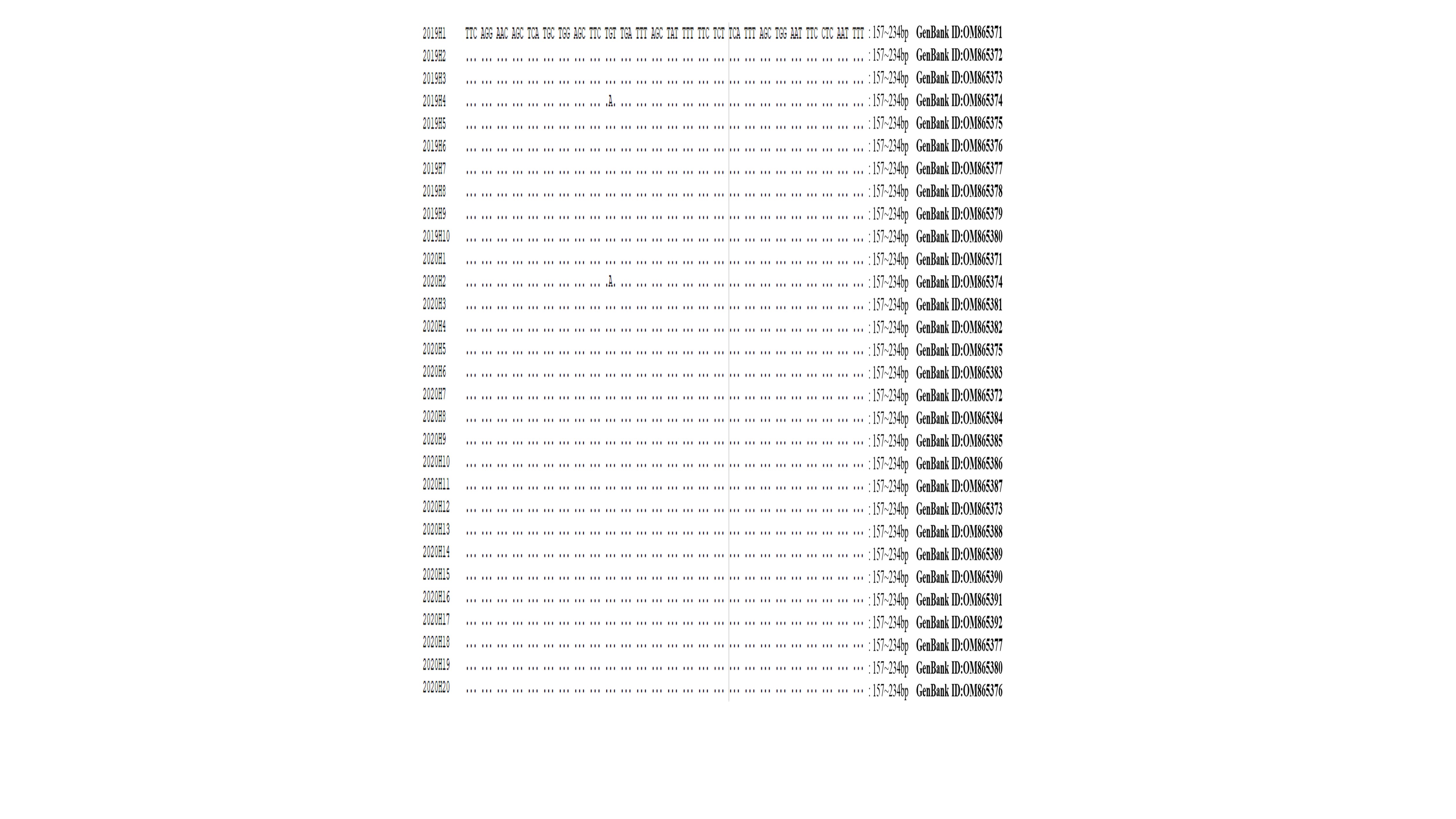

Supplement: Supplementary file 1 [file DataSheet1.ZIP › Appendix/Appendix 3. Haplotype information based on the mitochondrial COó± gene of Ae. aegypti/3.jpg]

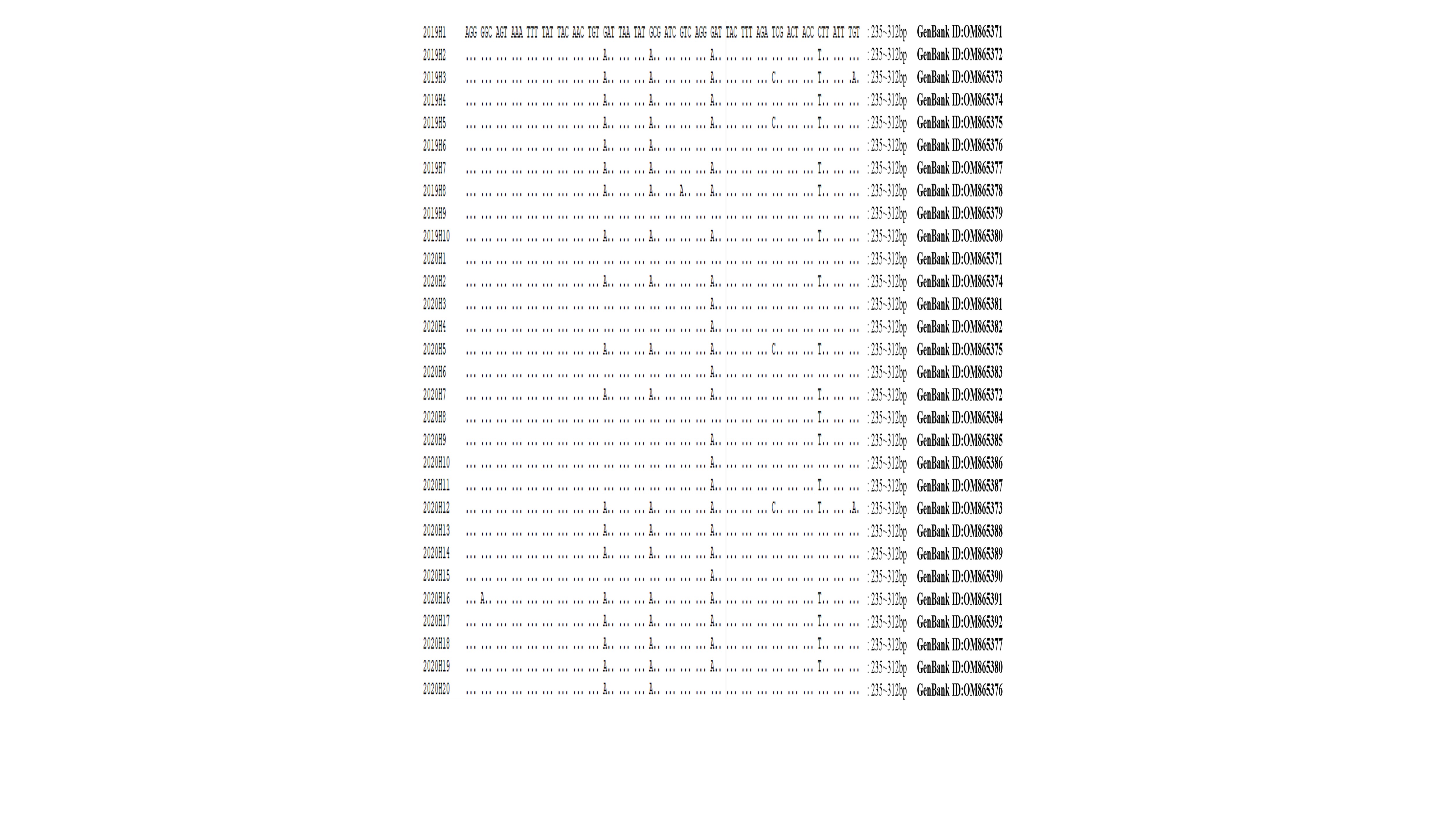

Supplement: Supplementary file 1 [file DataSheet1.ZIP › Appendix/Appendix 3. Haplotype information based on the mitochondrial COó± gene of Ae. aegypti/4.jpg]

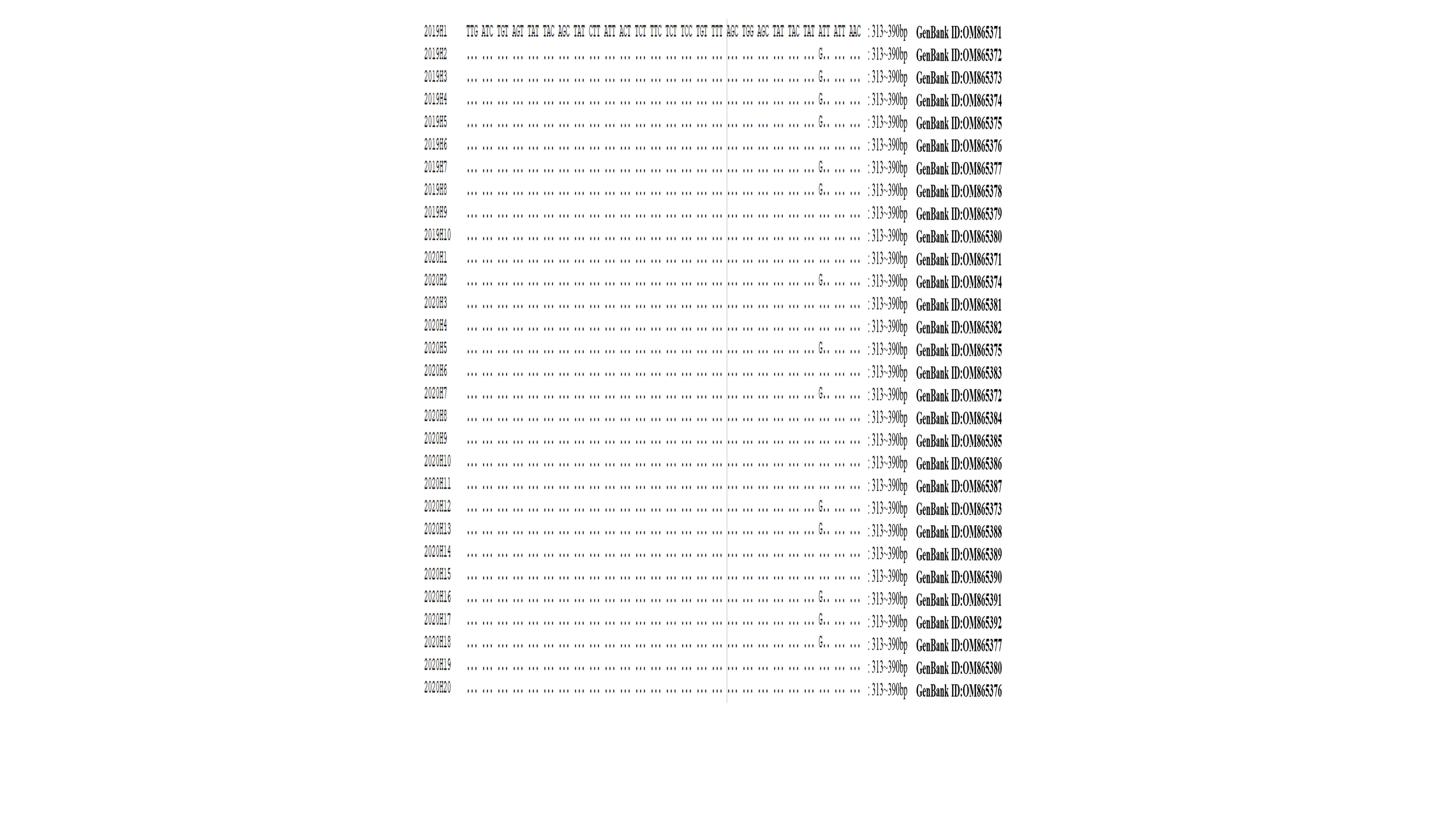

Supplement: Supplementary file 1 [file DataSheet1.ZIP › Appendix/Appendix 3. Haplotype information based on the mitochondrial COó± gene of Ae. aegypti/5.jpg]

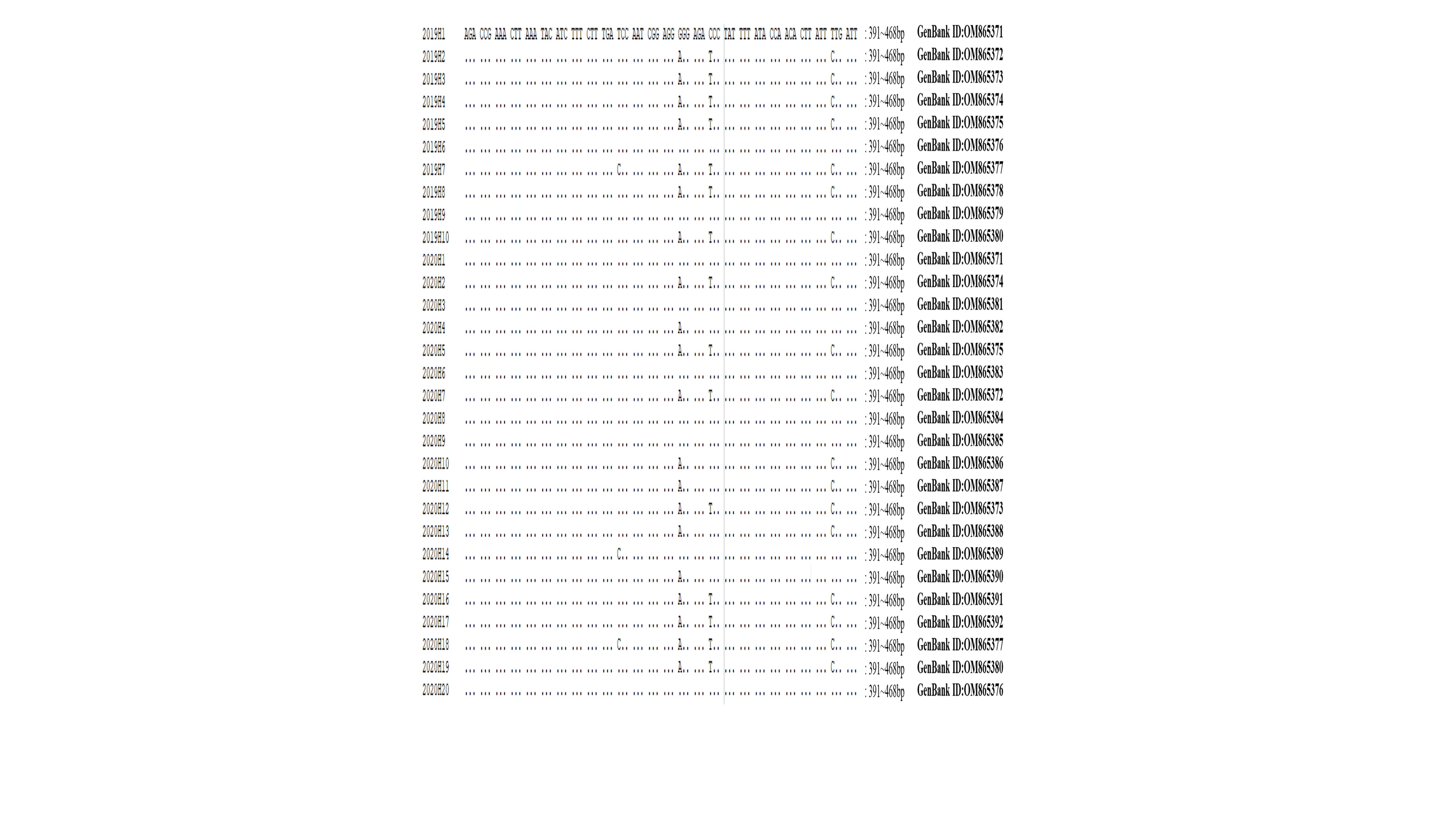

Supplement: Supplementary file 1 [file DataSheet1.ZIP › Appendix/Appendix 3. Haplotype information based on the mitochondrial COó± gene of Ae. aegypti/6.jpg]

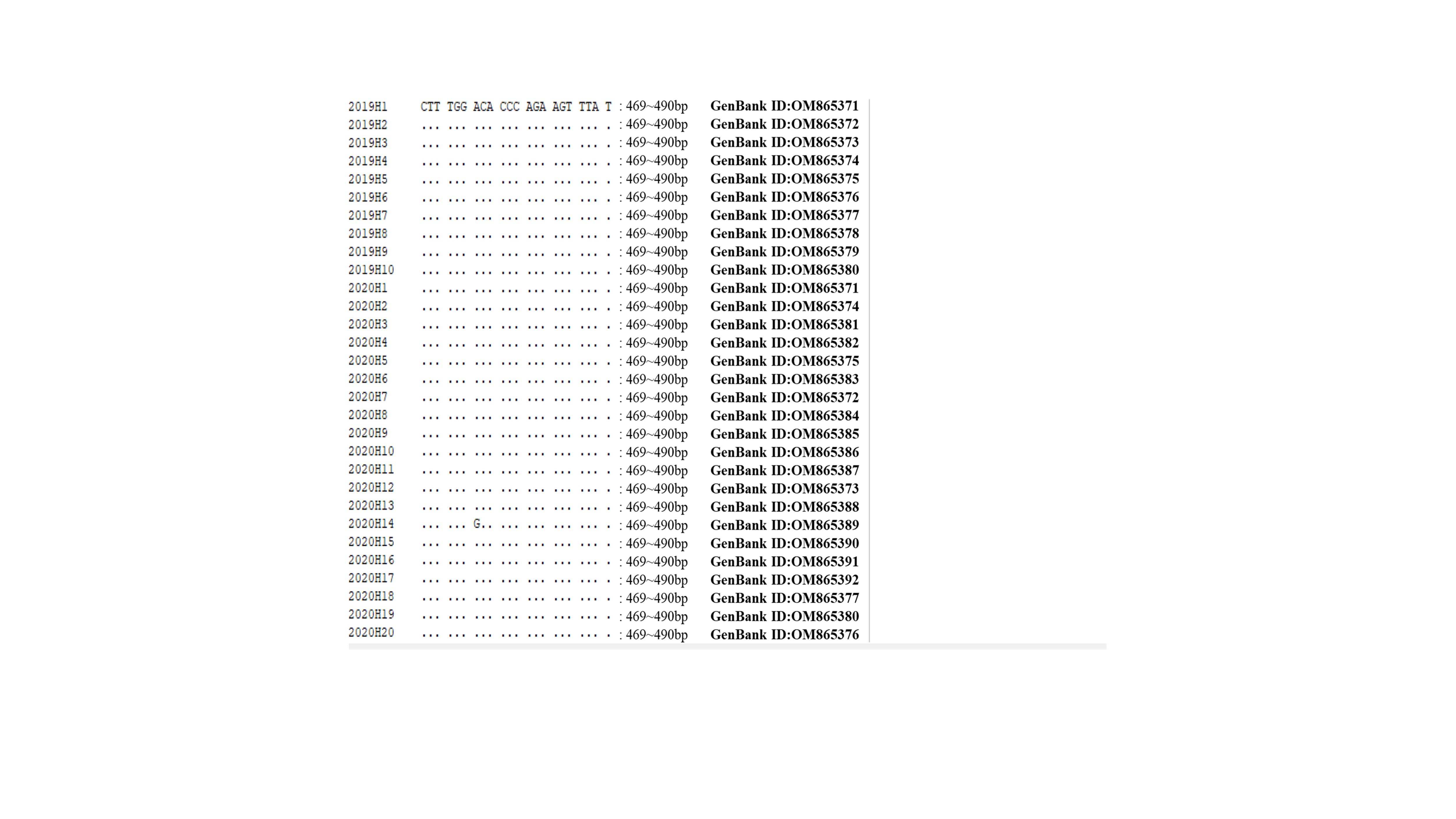

Supplement: Supplementary file 1 [file DataSheet1.ZIP › Appendix/Appendix 3. Haplotype information based on the mitochondrial COó± gene of Ae. aegypti/7.jpg]

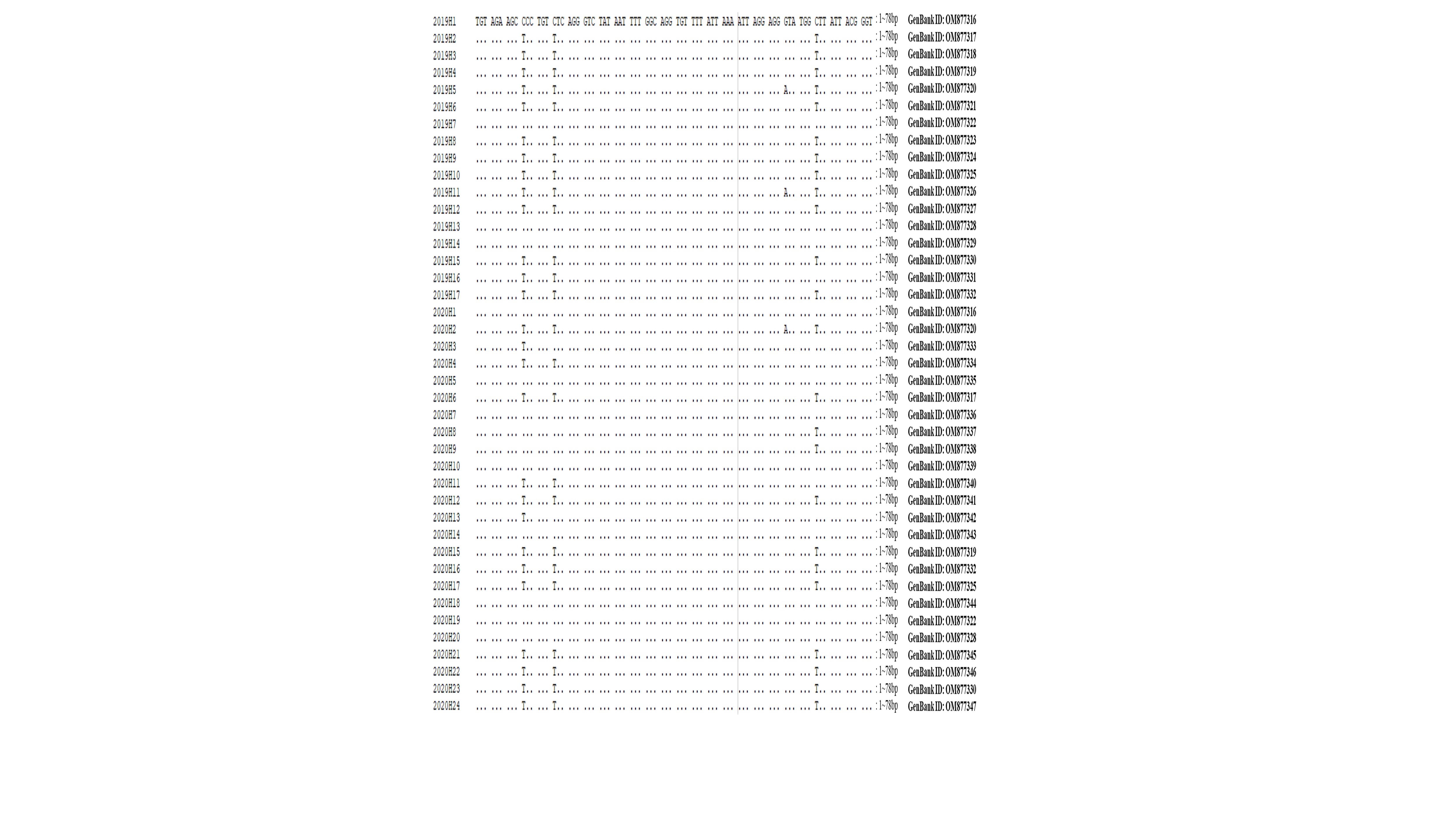

Supplement: Supplementary file 1 [file DataSheet1.ZIP › Appendix/Appendix 3. Haplotype information based on the mitochondrial ND4 gene of Ae. aegypti/1.jpg]

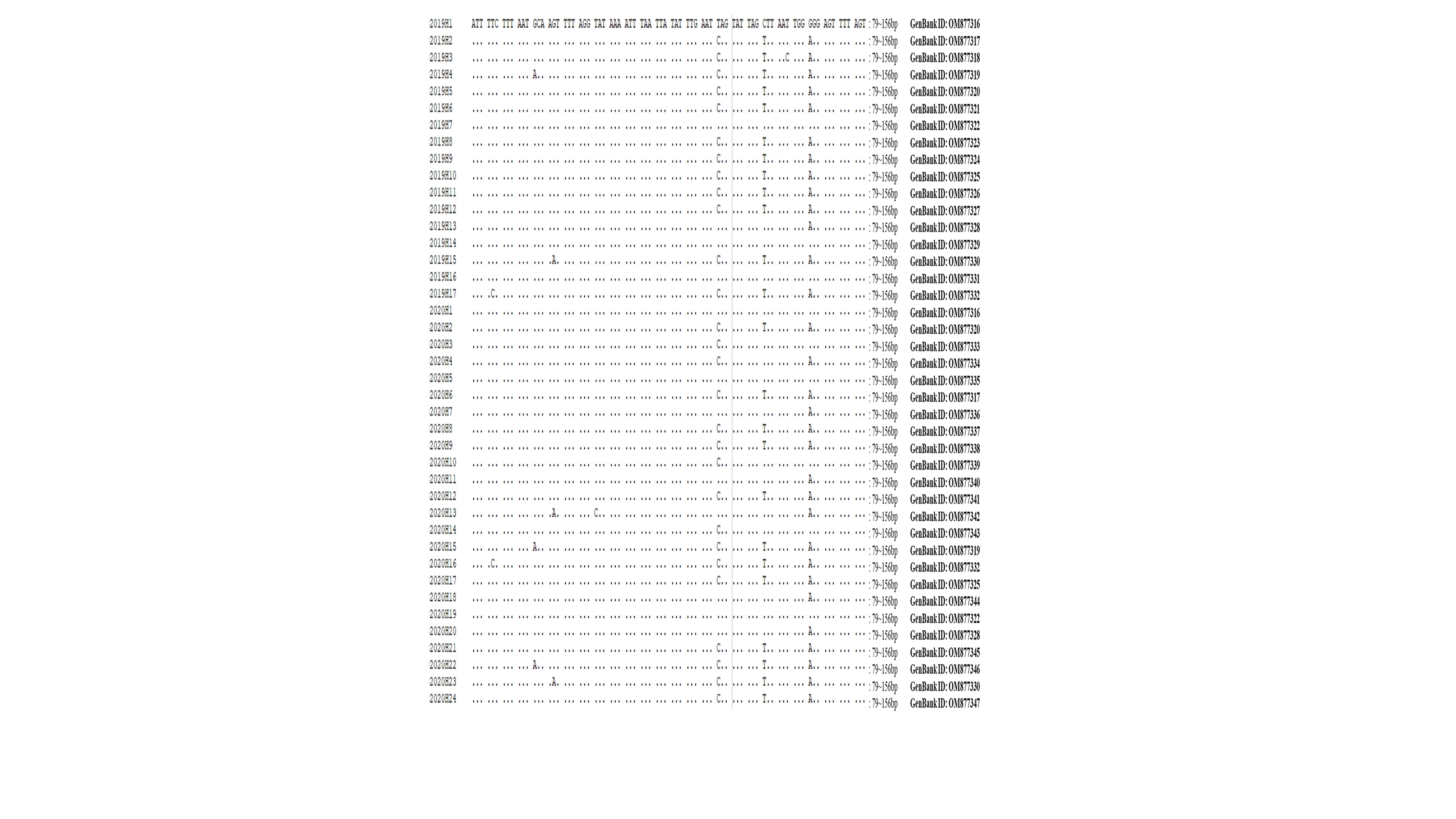

Supplement: Supplementary file 1 [file DataSheet1.ZIP › Appendix/Appendix 3. Haplotype information based on the mitochondrial ND4 gene of Ae. aegypti/2.jpg]

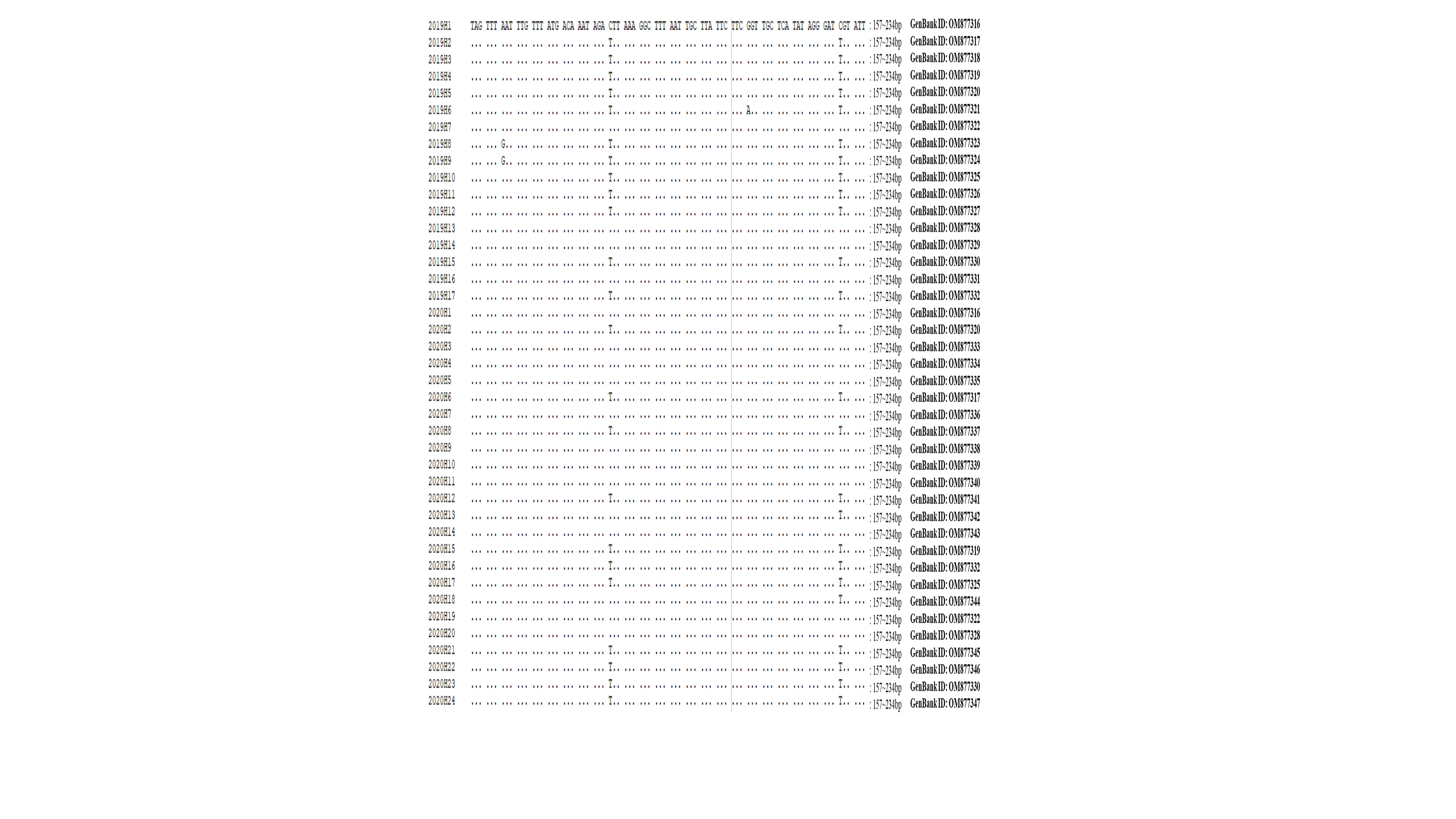

Supplement: Supplementary file 1 [file DataSheet1.ZIP › Appendix/Appendix 3. Haplotype information based on the mitochondrial ND4 gene of Ae. aegypti/3.jpg]

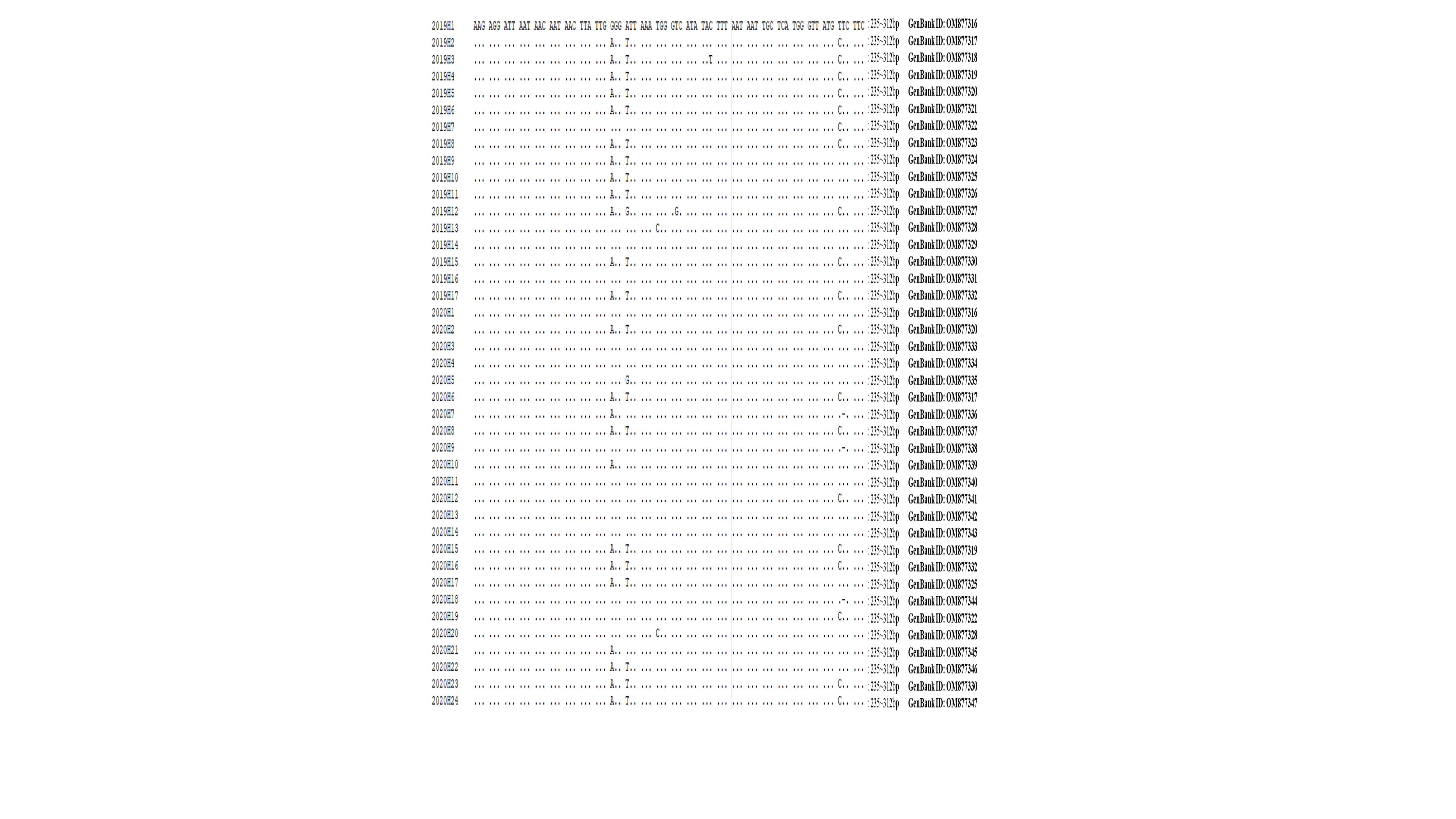

Supplement: Supplementary file 1 [file DataSheet1.ZIP › Appendix/Appendix 3. Haplotype information based on the mitochondrial ND4 gene of Ae. aegypti/4.jpg]

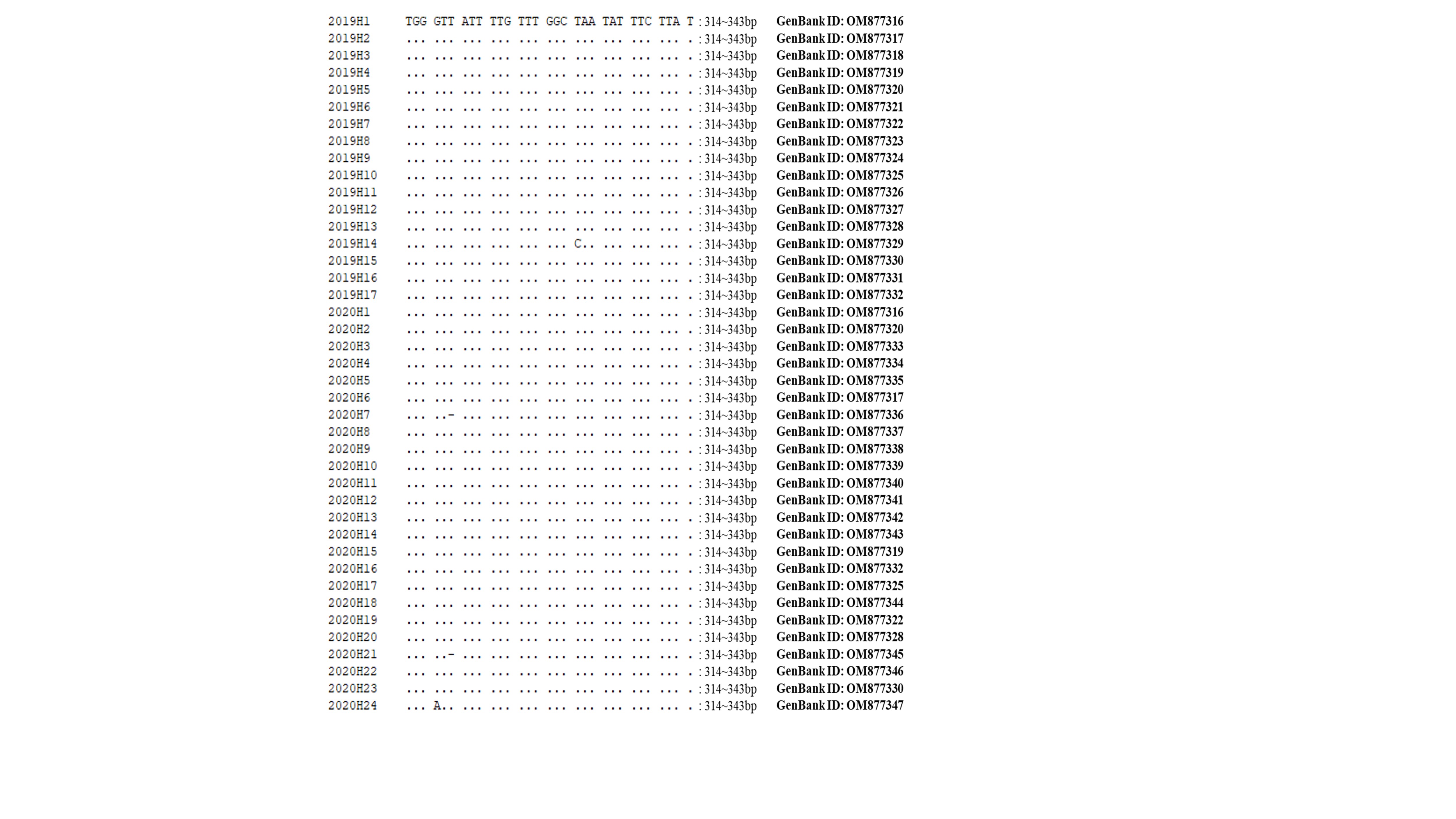

Supplement: Supplementary file 1 [file DataSheet1.ZIP › Appendix/Appendix 3. Haplotype information based on the mitochondrial ND4 gene of Ae. aegypti/5.jpg]
